# Characterization of kidney CD45^int^CD11b^int^F4/80^+^MHCII^+^CX3CR1^+^Ly6C^-^ “intermediate mononuclear phagocytic cells”

**DOI:** 10.1371/journal.pone.0198608

**Published:** 2018-06-01

**Authors:** Sul A. Lee, Sanjeev Noel, Mohanraj Sadasivam, Mohamad E. Allaf, Phillip M. Pierorazio, Abdel R. A. Hamad, Hamid Rabb

**Affiliations:** 1 Department of Medicine, Johns Hopkins University, Baltimore, Maryland, United States of America; 2 Yonsei University College of Medicine, Seoul, South Korea; 3 Department of Pathology, Johns Hopkins University, Baltimore, Maryland, United States of America; 4 Department of Urology, Johns Hopkins University, Baltimore, Maryland, United States of America; UCL Institute of Child Health, UNITED KINGDOM

## Abstract

Kidney immune cells play important roles in pathogenesis of many diseases, including ischemia-reperfusion injury (IRI) and transplant rejection. While studying murine kidney T cells, we serendipitously identified a kidney mononuclear phagocytic cell (MPC) subset characterized by intermediate surface expression of CD45 and CD11b. These CD45^int^CD11b^int^ MPCs were further identified as F4/80^**+**^MHCII^**+**^CX3CR1^**+**^Ly6C^-^ cells, comprising ~17% of total CD45^+^ cells in normal mouse kidney (*P* < 0.01) and virtually absent from all other organs examined except the heart. Systemic clodronate treatment had more significant depletive effect on the CD45^int^CD11b^int^ population (77.3%±5.9%, *P* = 0.03) than on CD45^high^CD11b^+^ population (14.8%±16.6%, *P* = 0.49). In addition, CD45^int^CD11b^int^ MPCs had higher phagocytic function in the normal kidney (35.6%±3.3% vs. 24.1%±2.2%, *P* = 0.04), but lower phagocytic capacity in post-ischemic kidney (54.9%±1.0% vs. 67.8%±1.9%, *P* < 0.01) compared to the CD45^high^CD11b^+^ population. Moreover, the CD45^int^CD11b^int^ population had higher intracellular production of the pro-inflammatory tumor necrosis factor (TNF)-α (58.4%±5.2% vs. 27.3%±0.9%, *P* < 0.001) after lipopolysaccharide (LPS) stimulation and lower production of the anti-inflammatory interleukin (IL)-10 (7.2%±1.3% vs. 14.9%±2.2%, *P* = 0.02) following kidney IRI, suggesting a functional role under inflammatory conditions. The CD45^int^CD11b^int^ cells increased early after IRI, and then abruptly decreased 48h later, whereas CD45^high^CD11b^+^ cells steadily increased after IRI before declining at 72h (*P* = 0.03). We also identified the CD45^int^CD11b^int^ MPC subtype in human kidney. We conclude that CD45^int^CD11b^int^ F4/80^**+**^MHCII^**+**^CX3CR1^**+**^Ly6C^-^population represent a unique subset of MPCs found in both mouse and human kidneys. Future studies will further characterize their role in kidney health and disease.

## Introduction

Both innate and adaptive immune mechanisms are important mediators of kidney injury and repair, and several different types of immune cells participate in these processes [1–3]. Resident mononuclear phagocytic cells (MPCs) in kidney serve sentinel roles in protection against pathogens and maintenance of homeostatic microenvironment [4, 5]. MPCs are functionally classified as either macrophages by their phagocytic role or as dendritic cells (DCs) by their antigen-presenting phenotype [6, 7]. Ontogenic similarities of macrophages and DCs and their functional/phenotypical heterogeneities have led to confusion during classification of MPCs, which makes it hard to use the traditional macrophage marker, F4/80, and the well-known DC marker, CD11c, to distinguish between these cell types [8–11]. Hence, despite recent advances in studying kidney MPC subpopulations and their functional characterization [12], their identification and classification remain incomplete [13].

Cell lineage markers, including CD11b and CD11c, are frequently used to discriminate MPCs from other immune cells and given the complexities and heterogeneities of MPCs in non-lymphoid organs, relative expression levels of CD11b and CD11c are often applied to distinguish between the MPC subpopulations. On the other hand, the level of CD45 expression has been used to discriminate between microglia and infiltrating macrophages in the central nervous system (CNS) [14–16]. Microglia, yolk sac-derived major resident macrophages in CNS, serve important role in homeostasis maintenance and recent studies have found that the resident microglia are functionally distinct from the myeloid-derived infiltrating macrophages [17]. However, the differential levels of CD45 expression among renal MPC populations have not been carefully studied to date. During flow cytometric analysis of lymphocytes in murine kidney, we serendipitously found an atypical cell population that was distinguishable from other immune cells by its intermediate CD45 expression. In the current study, we identified this population as a discrete renal MPCs that is absent in other organs except the heart and had unique phenotypic characteristics and functional properties, including cytokine production profiles and response to systemic clodronate treatment as well as to ischemia-reperfusion injury (IRI). We also identified this MPC population in “normal” human kidney samples from patients undergoing nephrectomies for renal cell carcinoma (RCC), extending the relevance of our mouse findings to humans.

## Materials and methods

### Animals

Male C57BL/6 wildtype (WT) mice were purchased from the Jackson Laboratory (ME, USA) and kept under specific pathogen-free conditions at the central animal facility of the Johns Hopkins University. Male mice 8–10 weeks of age were used and all animal experiments were performed using Johns Hopkins University Institutional Animal Care and Use Committee-approved protocols. (Protocol number: M016M71)

### Preparation of single cells from kidney and other organs

We used an established protocol for kidney mononuclear cell isolation [18]. Briefly, mice were anesthetized with intraperitoneal ketamine hydrochloride (130 mg/kg) and xylazine (7 mg/kg), underwent midline abdominal incisions, and were exsanguinated. Both kidneys were removed after renal pedicle dissection and decapsulated. For the enzymatic digestion (ED) process, kidneys were finely minced and incubated in collagenase D (2 mg/ml; Sigma-Aldrich, St. Louis, MO, USA) solution for 30 minutes at 37°C. Single cell suspension of the kidney digestion were achieved by mechanical disruption of the tissue using a 70 μm strainer (BD bioscience). Mechanical digestion (MD) alone were used for the comparison between ED and MD for kidney digestion by omitting the step of collagenase incubation. No difference in cell viability was found between ED and MD (**S1 Fig**).

Each lymphoid organ was filtered through 40 μm strainer (BD bioscience) to prepare single cell suspensions and spleen was incubated with erythrolysis buffer. Single cell suspension from non-lymphoid organs were prepared based on previously published techniques [19–22]. First, organs were removed after systemic perfusion. Peyer’s patches and epithelium-containing supernatants were separated from small intestine. Heart, lung, liver and remnant intestine tissues were cut into 1–2 mm sized pieces and incubated for 30 minutes in 2 mg/ml of collagenase D solution at 37°C. Digested tissues were filtered using a 70 μm strainer. After washing, cell suspensions from non-lymphoid organs were then run at room temperature with isotonic Percoll density (GE Healthcare, Chicago, IL, USA) centrifugation (1,500 x g for 30 minute in brake off mode) to collect mononuclear cell population, as per the manufacturer’s instructions. Collected cells from each organ were washed and each pellet was re-suspended in RPMI media containing 5% FBS and counted.

### Antibodies and reagents

All reagents used for media and PBS were obtained from Sigma-Aldrich and BD Bioscience. Fluorochrome-conjugated anti-mouse mAbs including anti-CD45 (30-F11; Rat IgG2b, κ) APC-Cy7/BUV395, anti-CD11b (M1/70; Rat IgG2b, κ) eFluor 450, anti-CD11c (N418; Armenian Hamster IgG) PE-Cy7, anti-F4/80 (BM8; Rat IgG2a, κ) APC, anti-Ly6C (HK1.4; Rat IgG2c, κ) PE, anti-Ly6G (IA8; Rat IgG2a, κ) FITC, anti-CCR7 (4B12; Rat IgG2a, κ) PerCP-Cy5.5, anti-CD206 (C068C2; Rat IgG2b, κ) PE, anti-CD103 (2E7; Armenian Hamster IgG) PerCP-Cy5.5, anti-CD19 (1D3; Rat IgG2a, κ) FITC, anti-TCRβ (H57-597; Armenian Hamster IgG) FITC, anti-γδTCR (eBioGL3; Armenian Hamster IgG) FITC, anti-CD3 (17A2, IgG2b, κ), anti-CD4(RM4-5; Rat IgG2a, κ) APC, anti-CD8α (53–6.7; Rat IgG2a, κ & 5H10-1; Rat IgG2b, λ) PerCP-Cy5.5, anti-CD8β (eBioH35-17.2; Rat IgG2b, κ & YTS156.7.7; Rat IgG2b, κ) FITC, anti-CD49b (DX5; Rat IgM, κ) FITC, anti-NK1.1 (PK136; Mouse IgG2a, κ) FITC, anti-interferon (IFN)-γ (XMG1.2; Rat IgG1, κ) PerCP-Cy5.5, anti-tumor necrosis factor (TNF)-α (MP6-XT22; Rat IgG1, κ) PE, anti-interleukin (IL)-10 (JES5-16E3; Rat IgG2b, κ) PE, anti-IL-4 (11B11; Rat IgG1, κ) PE-Cy7, anti-Ki-67 (SolA15; Rat IgG2a, κ) APC, and biotin-conjugated anti-CD16/32 (2.4G2; Rat IgG2b, κ) and anti-MHCII^IA-b^ (AF6-120.1; Mouse IgG2a, κ) were from BD Biosciences, eBioscience (San Diego, CA, USA), or BioLegend and used at 1:50 to 1:200 dilution. When samples were stained with biotin-conjugated mAbs, cells were washed with FACS buffer twice and followed by staining with streptavidin-conjugated PE-Cy7 or APC-Cy7.

### Intracellular cytokine staining

For intracellular cytokine staining, single-cell suspensions of isolated kidney mononuclear cells (KMNCs) from normal or post-ischemic mice were incubated for 6 hours at 37°C in a 5% CO_2_ humidified atmosphere incubator in the presence of Golgi Plug (BD Biosciences) with or without innate activator lipopolysaccharide (LPS) (100 ng/ml). After surface staining for 30 minutes at 4°C, cells were then permeabilized with fixation/permeabilization buffer (eBioscience) for 30 minutes followed by an additional 10 minute incubation with FcR blockers and intracellular cytokine staining with monoclonal antibodies including IFN-γ, TNF-α, IL-10, and IL-4 for 30 minutes. Unstimulated MPCs from normal mice were analyzed together as a negative control.

### Flow cytometry

Cell pellets were resuspended in ice-cold FACS buffer (PBS containing 1% BSA and 0.01% sodium azide) and preincubated with anti-CD16/CD32 Fc receptor (FcR) for 10 minutes on ice. Cells were then incubated with Ab mixture for 30 minutes at 4°C, washed with FACS buffer, and subsequently analyzed. Multi-color immunofluorescence staining was analyzed with LSR II using FACS Diva software (BD Biosciences) and analyzed using FlowJo software (Versions 10.2). Initially, polygon gate was applied to size scatter gate excluding debris and dead cells, followed by single cell gates. Kidney MPCs were analyzed based on the expression of CD45 and CD11b after excluding any cells expressing Ly6G, CD19, αβTCR, γδTCR nor CD49b using dump channel (**S2 Fig**).

### Proliferation assays

Proliferation of each MPC population after IRI was assessed by measuring intracellular expression of Ki-67 antigen using anti-Ki-67 mAb. KMNCs were isolated from kidney and stained for surface markers, followed by intracellular staining of Ki-67 using Foxp3/Transcription Factor Staining Buffer Set (eBioscience). Samples were acquired by LSR II using FACS Diva software and analyzed as described earlier.

### Apoptosis assays

Apoptosis was assessed using PE-Annexin V apoptosis kit (BD Biosciences) according to manufacturer’s instruction. KMNCs were isolated from kidney and stained for surface markers, followed by re-suspension in Annexin V binding buffer with PE-labeled Annexin V for 15 min in dark at room temperature. Samples were acquired by flow cytometer and analyzed as described earlier.

### Bone marrow isolation and macrophage enrichment

The hind limb long bones (femur and tibia) were dissected from euthanized mice. After metaphysis exposure, bone marrow was harvested via centrifugation [23]. Isolated bone marrow cell pellets were dissociated in 5 ml erythrolysis buffer for 1 min then washed with 20 ml of ice-cold 5% RMPI buffer. After centrifugation, cells were suspended in 5% RPMI media and transferred to a 100 mm sterile culture dish. After 2 hour incubation at 37°C in a 5% CO_2_ humidified atmosphere incubator, supernatant was aspirated and discarded and adherent macrophages were harvested after 10 minute incubation in ice-cold PBS. Cells were then incubated in 2 mg/ml of collagenase D solution or 5% RPMI media for 30 minutes at 37°C. Control cells were incubated in 5% RPMI media without collagenase D.

### Administration of liposome-encapsulated clodronate

Liposomal clodronate and PBS-encapsulated control liposomes were purchased from Encapsula NanoSciences (Brentwood, TN, USA). Mice were injected intraperitoneally with 100 μl of liposomal clodronate or control liposomes twice with 48 hour interval and were used for experiments 24 hours after the second dose.

### Phagocytosis assay

Isolated KMNCs from normal or post-ischemic mice were incubated in RPMI media including 10% FBS, 5mM HEPES, 100 IU/ml penicillin and streptomycin, with latex beads-PE complex in Phagocytosis Assay Kit (Cayman Chemical Company, Ann Arbor, MI, USA) for 2 hours. After further staining with surface markers, cells were analyzed using flow cytometry. PE fluorescent signal from T lymphocytes were also analyzed as a negative control.

### Mouse renal ischemia-reperfusion (IRI) model

An established model of renal ischemia-reperfusion in mice was used [24, 25]. Briefly, mice were anesthetized with an intraperitoneal injection of ketamine hydrochloride (130 mg/kg) and xylazine (7 mg/kg). Following an abdominal medial incision, the renal pedicle was dissected, and a microvascular clamp (Roboz Surgical Instrument, Gaithersburg, MD) was placed on each renal pedicle for 30 minutes. Animals were kept hydrated with 1 ml of warm saline and at a constant temperature (37°C). After 30 minutes of ischemia, the clamps were removed and the kidneys were inspected for restoration of blood flow. The wounds were sutured and the animals were allowed to recover with free access to food and water.

### Human samples

The present study was conducted in accordance with the Declaration of Helsinki and approved by the Johns Hopkins Medicine Institutional Review Boards. Kidney samples were collected from RCC patients undergoing partial or total nephrectomies after getting informed written consent from each patient. No subject identifiable information was acquired during tissue collection. The kidney tissue was digested according to our protocol for isolation of KMNCs; stained with mAb anti-CD45 (H130) APC-Cy7 (BioLegend, San diego, CA, USA), anti-CD11b (ICRF-44) Pacific blue, (BD Biosciences, Franlkin Lakes, NJ, USA) and anti-CD15 (HI98) FITC (BioLegend). Similarly, polygon gate was applied to size scatter gate excluding debris and dead cells, followed by single cell gates. Kidney MPCs were analyzed based on the expression of CD45 and CD11b after excluding CD15^+^ cells.

### Statistics

Statistical differences were analyzed using two-tailed Student t test between two groups and one-way ANOVA test for three or more groups (GraphPad Software, La Jolla, CA). Statistical significance was determined as *P* < 0.05.

## Results

### CD45^int^CD11b^int^ population constitutes a significant component of the kidney immune cells

**Fig 1:**
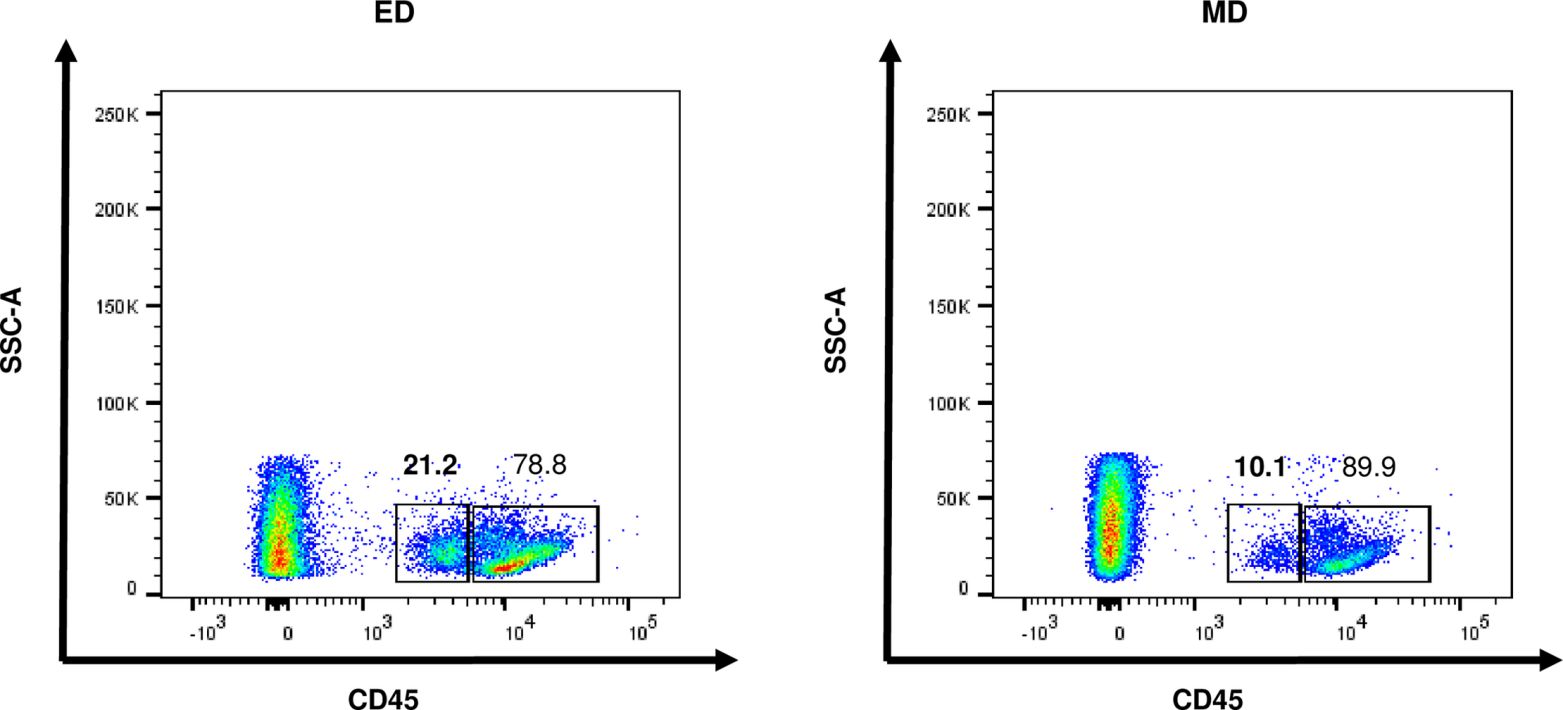
CD45^int^ population are present in normal mouse kidney. Representative plots show CD45^int^ cells and CD45^high^ cells in normal mouse kidneys. ED improves the identification of CD45^int^ population compared to MD alone. Data are from one of 11 representative experiments with similar results. ED, enzymatic digestion; MD, mechanical digestion.

While studying TCRαβ ^**+**^CD4^-^CD8^-^kidney αβ T cells in mouse and human kidneys using standard flow cytometry gating technique (**see** Materials and methods **and S3 Fig**), we observed two clearly distinct CD45^high^ and CD45^int^ populations (**Fig 1**) that were identifiable even after using different concentrations of CD45 mAb. In addition, use of ED significantly increased the proportion of CD45^int^ KMNCs as compared to MD alone (21.2% vs. 10.1% among CD45^**+**^cells).

**Fig 2:**
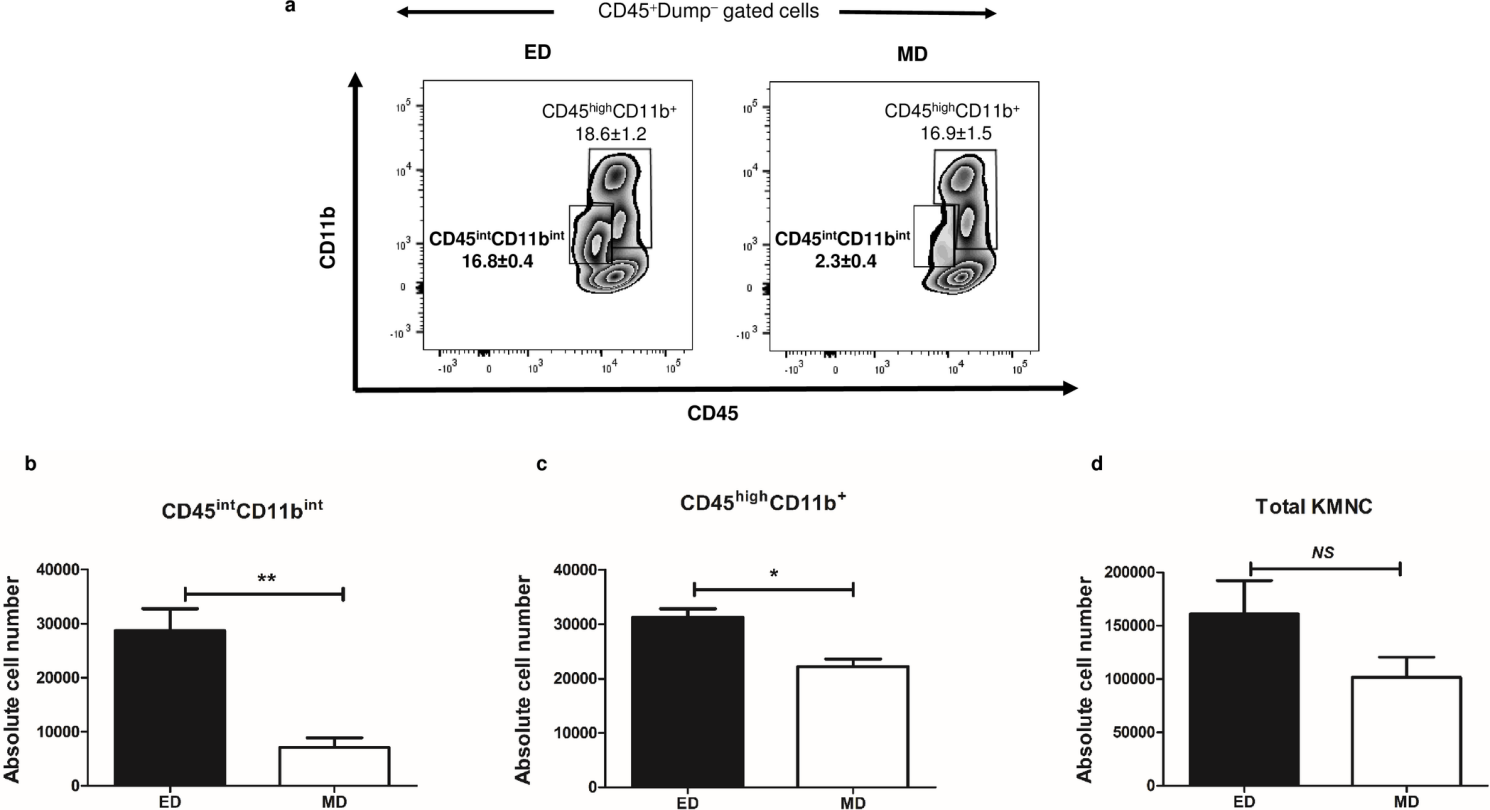
CD45^int^CD11b^int^ cells are distinct mononuclear phagocytic population in normal mouse kidney. (a) Representative plots show that ED yields a greater proportion of CD45^int^CD11b^int^ cells compared to MD alone in normal mice kidney. Numbers on plots represent the percentage of each population among CD45^**+**^ population. (b**-**d) Bar graph shows absolute cell number of each population isolated by ED or MD method. The use of ED significantly increased the absolute number of the CD45^int^CD11b^int^ population (b) and CD45^high^CD11b^**+**^ population (c), but there was no significant difference in the total number of KMNCs (CD45^**+**^ population) (d). Data are displayed as means ± SEM (n = 3/group). ED, enzymatic digestion; MD, mechanical digestion. *P < 0.05; **P < 0.01; *NS*, no statistically significant difference between groups.

Further characterization showed that the CD45^int^ population were positive for CD11b expression, but not for lineage markers of lymphocytes, neutrophils or NK/NKT cells. (**S4 Fig**). Consequently, we excluded lymphocytes, neutrophils or NK/NKT cells using a dump channel, gated the remaining CD45^**+**^ cells, and further analyzed for CD11b expression (**see** Materials and methods **and S2 Fig**). This gating strategy clearly revealed a distinct population that expressed intermediate levels of CD45 and CD11b (**Fig 2A**). This is hereafter referred to as renal CD45^int^CD11b^int^ population comprised 16.8%±0.4% of total CD45^**+**^ cells. Another population of kidney MPCs expressed high levels of CD45 with mid-to high levels of CD11b and referred to as CD45^high^CD11b^**+**^ population, consisting 18.6%±1.2% of total CD45^**+**^ cells. Effects of the two digestion methods were assessed again and use of ED significantly increased the absolute number of CD45^int^CD11b^int^ population as compared to MD alone (2.8x10^4^±0.4x10^4^ vs. 0.7x10^4^±0.2x10^4^, *P* < 0.01) (**Fig 2B**). Isolation of CD45^high^CD11b^**+**^ cells were also significantly improved with ED compared to MD alone (3.1x10^4^±0.2x10^4^ vs. 2.2x10^4^±0.1x10^4^, *P* = 0.02) (**Fig 2C**). On the other hand, the total number of KMNCs was not affected by these digestion methods (1.6x10^5^±0.3x1^5^ vs. 1.0x10^5^±0.2x10^5^, *P* = 0.07). (**Fig 2D**). We ruled out that low expression of CD45 and CD11b is due to collagenase treatment because treatment of bone marrow CD45^high^CD11b^high^ cells with collagenase for 30 minutes did not lower their expression levels (**S5 Fig**).

### Characterization of CD45^int^CD11b^int^ mononuclear phagocytic cells in kidney using monocyte-, macrophage-, dendritic cell-associated surface markers

**Fig 3:**
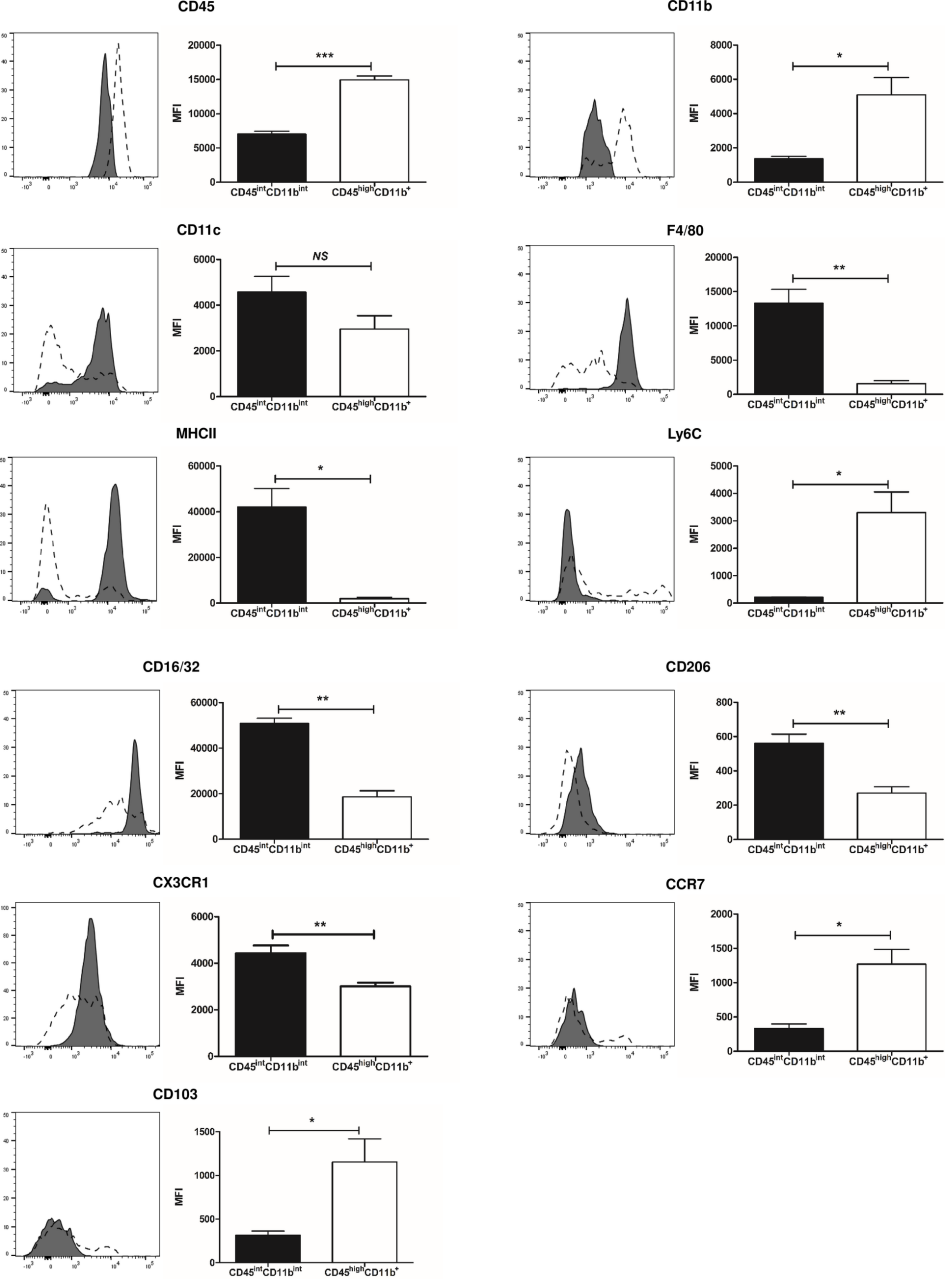
CD45^int^CD11b^int^ MPCs have different phenotype compared to CD45^high^CD11b^+^ MPCs. (left) Representative histograms of indicated markers. Filled plot shows expression level of each markers by CD45^int^CD11b^int^ MPCs and dashed plot shows one from CD45^high^CD11b^**+**^ cells. (right) Graphs show mean fluorescence intensity (MFI) of each indicated markers. Data are displayed as means ± SEM (n = 3-5/group). *P < 0.05; **P < 0.01; ***P < 0.001; *NS*, no statistically significant difference between groups.

The CD45^int^CD11b^int^ population was further characterized using monocyte-, macrophage-, dendritic cell-associated markers. As shown in **Fig 3**, this CD45^int^CD11b^int^ population had higher expression of F4/80, MHCII, CD16/32, CD206 and CX3CR1 and lower expression of CD45, CD11b, Ly6C, CCR7 and CD103 compared to the CD45^high^CD11b^**+**^ population. The percent expression of F4/80, MHCII and CX3CR1 was over 95% whereas that of Ly6C expression was below 5%. These data identified CD45^int^CD11b^int^ populations as F4/80^**+**^MHCII^**+**^CX3CR1^**+**^Ly6C^−^ MPCs. Expression of CD11c was also relatively high among CD45^int^CD11b^int^ population, ranging from 70–90%.

Co-expression of F4/80 and CD11c, the traditional macrophage and DC marker, respectively, has been found in renal MPC populations in the previous studies and the phenotypical and functional characteristics of DCs and macrophages significantly overlap each other in kidney [26]. Also, there is lack of definitive surrogate marker for the clear distinction of renal macrophages and DCs in kidney [8]. For these reasons, CD45^int^CD11b^int^ population was further studied based on the spectrum of MPCs without further discrimination into macrophages or DCs.

### CD45^int^CD11b^int^ MPCs reside selectively in the kidney and to lesser extent in the heart but not in other lymphoid and non-lymphoid organs

**Fig 4:**
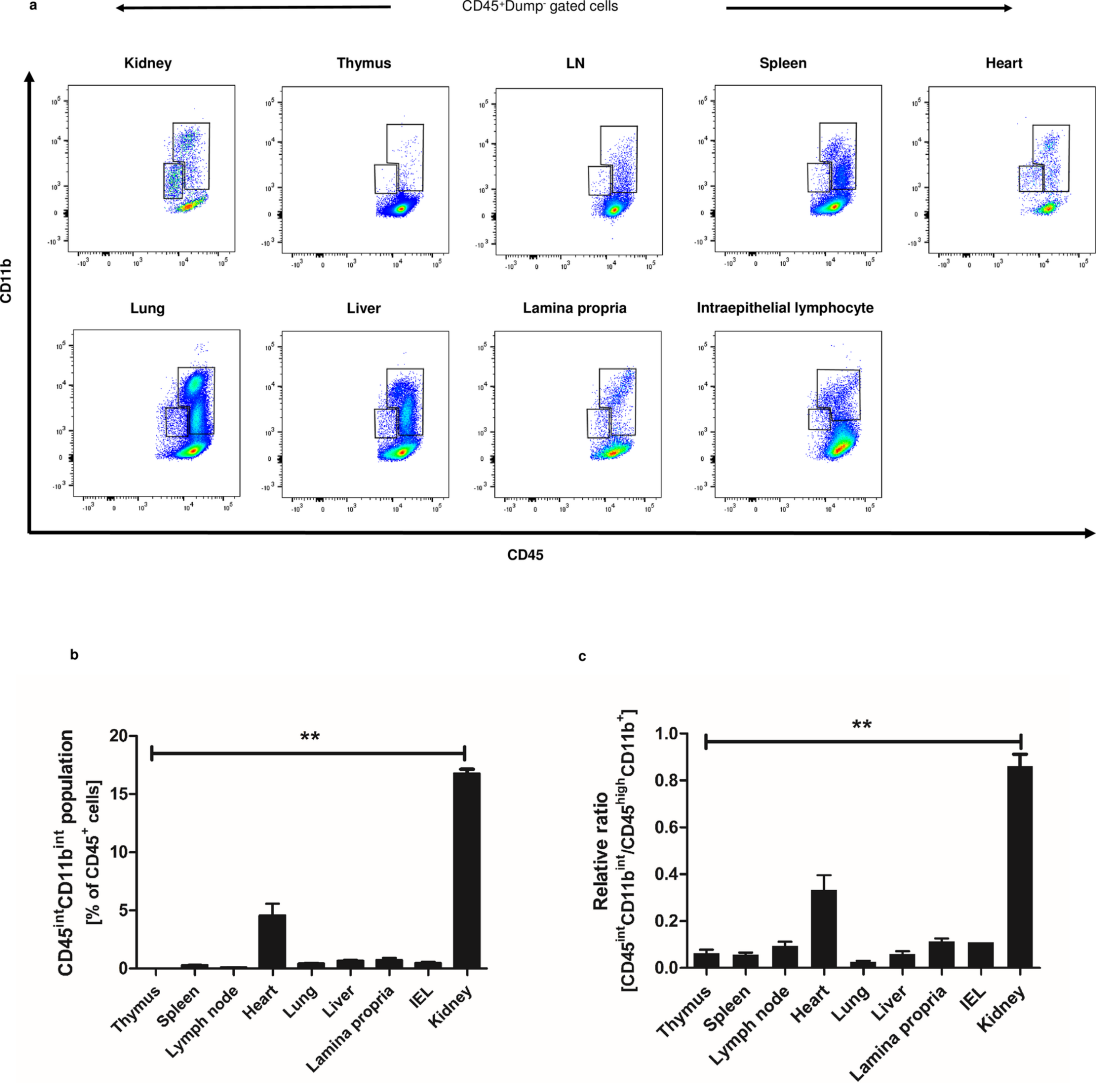
CD45^int^CD11b^int^ MPCs are predominately found in the kidney compared to other lymphoid and non-lymphoid organs. (a) Representative CD45 vs. CD11b plot of mononuclear cells from lymphoid and non-lymphoid organs of 8–10 week-old C57BL/6 wildtype male mice. (b) Bar graph shows the proportion of CD45^int^CD11b^int^ population among total CD45^**+**^ cells in each organ. (c) Relative ratios of CD45^int^CD11b^int^ cells over CD45^high^CD11b^**+**^ population in each organ are shown here. Data are displayed as means ± SEM (n = 3/group). IEL, intraepithelial lymphocyte. **P < 0.01.

The characteristic surface phenotype of kidney CD45^int^CD11b^int^ cells led us to search for their presence among MPCs in other organs. Most CD45^**+**^Dump^**-**^ cells in all other organs examined expressed high levels of CD45 (**Fig 4A**) with CD45^int^CD11b^int^ MPCs constituting less than 1% of total CD45^**+**^ cells except in the heart where CD45^int^CD11b^int^ MPCs comprised 4.5%±1.0% of CD45^**+**^ cells (**Fig 4B**). In contrast, kidney CD45^int^CD11b^int^ MPCs comprised 16.8%±0.4% of total CD45^**+**^ cells (*P* < 0.01) and the relative ratio of CD45^int^CD11b^int^ MPCs to CD45^high^CD11b^**+**^ cells reached 0.8±0.1 (*P* < 0.01) (**Fig 4C**). These results demonstrate that CD45^int^CD11b^int^ MPCs represent a major subset of MPCs in murine kidneys.

### Kidney CD45^int^CD11b^int^ MPCs decrease more significantly in response to systemic clodronate injection

**Fig 5:**
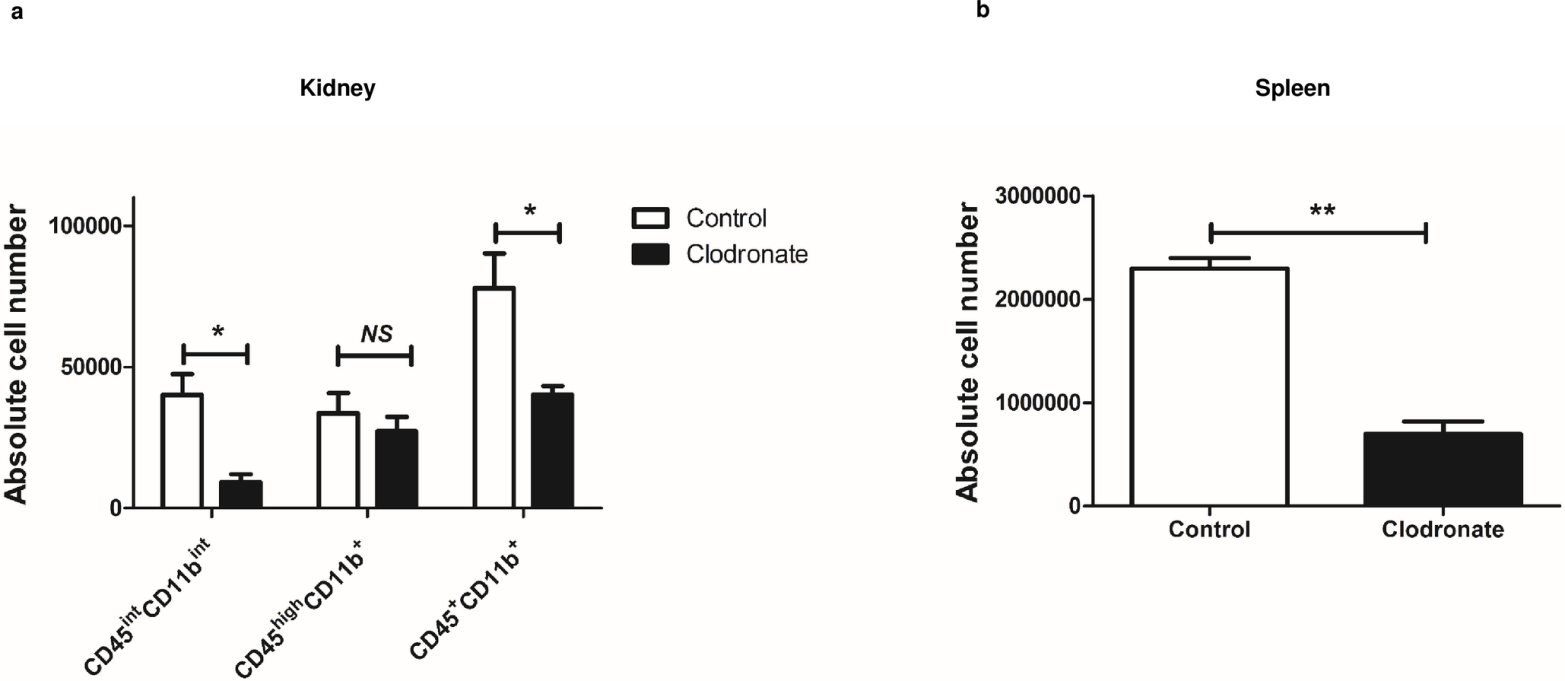
Systemic administration of liposomal clodronate has more pronounced depletive effects on CD45^int^CD11b^int^ population. (a) Bar graph shows absolute cell numbers in each population after systemic administration of liposomal clodronate or control liposome in normal mouse kidney. The effect of systemic clodronate is more prominent in CD45^int^CD11b^int^ population compared to CD45^high^CD11b^**+**^ or total CD45^**+**^CD11b^**+**^. (b) Bar graph shows absolute cell numbers of CD45^**+**^CD11b^**+**^ cells after systemic administration of liposomal clodronate or control liposome in wild-type mouse spleen. Data are displayed as mean ± SEM (n = 3/group). *P < 0.05; **P < 0.01; *NS*, no statistically significant difference between groups.

We used liposome clodronate to determine whether CD45^int^CD11b^int^ cells are bona fide myeloid cells with phagocytic functions. Liposomal clodronate is a phagocyte-ablating agent which induces apoptosis when phagocytosed by macrophages or dendritic cells [27]. Thus, we used it to assess phagocytic function of CD45^int^CD11b^int^ cells. Systemic injection of liposomal clodronate caused over 50% depletion of total CD45^**+**^CD11b^**+**^ cells in the kidney, and particularly ablated CD45^int^CD11b^int^ (77.3%±5.9% decrease, *P* = 0.03) compared to CD45^high^CD11b^**+**^ cells (14.8%±16.6% decrease, *P* = 0.49) (**Fig 5A**). Spleens of injected mice were analyzed in parallel and used as a positive control, but CD45-based subgrouping was not used here due to the sparsity of CD45^int^ cells in the spleen. Spleen CD45^**+**^CD11b^**+**^ population decreased 69.9%±4.7% in treated mice (*P* < 0.01), confirming the phagocyte-ablating effect of clodronate (**Fig 5B**).

### Kidney CD45^int^CD11b^int^ MPCs show variable phagocytosis capacity depending on the mode of stimulation

**Fig 6:**
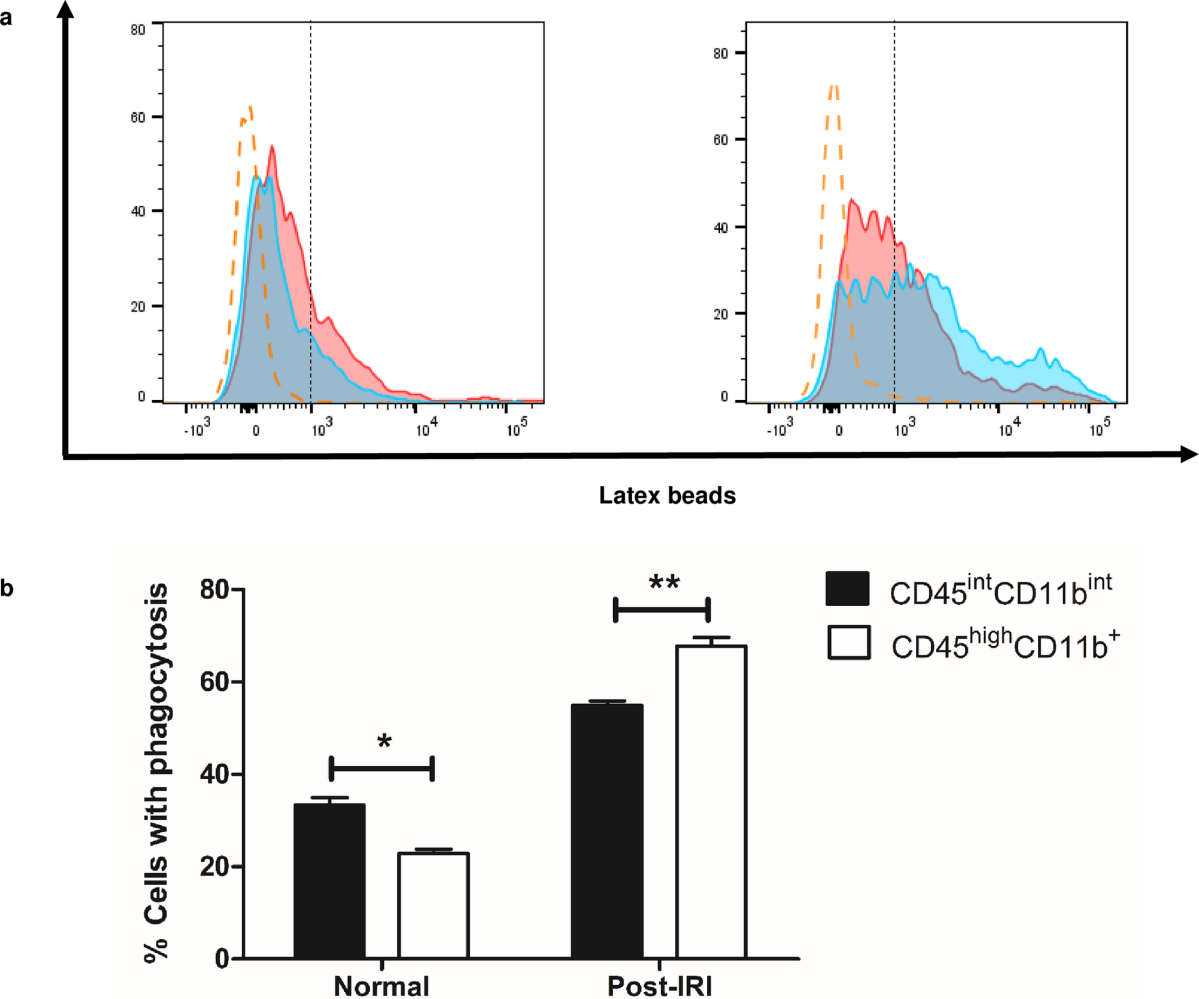
Comparison of phagocytic function between CD45^int^CD11b^int^ MPCs and CD45^high^CD11b^+^ MPCs. (a) Representative histograms for the uptake of fluorescent beads by different kidney MPCs from 8–10 week-old C57BL/6 wild type male mice in steady status (left), and in post-ischemic injury (right). Red plot shows the level of phagocytosed latex beads by CD45^int^CD11b^int^ MPCs and blue plot represents one by CD45^high^CD11b^+^ population. Yellow dashed line indicates the PE signal from T lymphocytes, being used as a control. (b) Bar graphs show the percentage of cells with the phagocytosed fluorescence beads in each population. Data are displayed as means ± SEM (n = 3/group). *P < 0.05; **P < 0.01.

In light of the higher depletive effect of liposomal clodronate on the CD45^int^CD11b^int^ population, we moved to an *ex vivo* phagocytosis assay to further compare the phagocytic functions of the CD45^int^CD11b^int^ and CD45^high^CD11b^+^ populations in the steady state and under IRI. Latex beads coated with fluorescently-labeled rabbit IgG was used as a probe for the assessment of phagocytic function and the engulfed fluorescent beads were analyzed by flow cytometry in the respective population. CD45^int^CD11b^int^ MPCs exhibited higher phagocytic function than the CD45^high^CD11b^**+**^ population (35.6%±3.3% vs. 24.1%±2.2%, *P* = 0.04) in normal mice. The phagocytic function of both populations increased after IRI compared to steady status. Unexpectedly, however, the phagocytic capacity of CD45^int^CD11b^int^ MPCs was significantly lower than that of the CD45^high^CD11b^+^ MPCs (54.9%±1.0% vs. 67.8%±1.9%, *P* < 0.01) after IRI (**Fig 6**). These data clearly show that CD45^int^CD11b^int^ cells have phagocytic function that surpasses or trails that of CD45^high^CD11b^+^ population depending on the micro-environmental conditions.

### Kidney CD45^int^CD11b^int^ MPCs have distinctive cytokine profiles in response to LPS stimulation or kidney IRI

**Fig 7:**
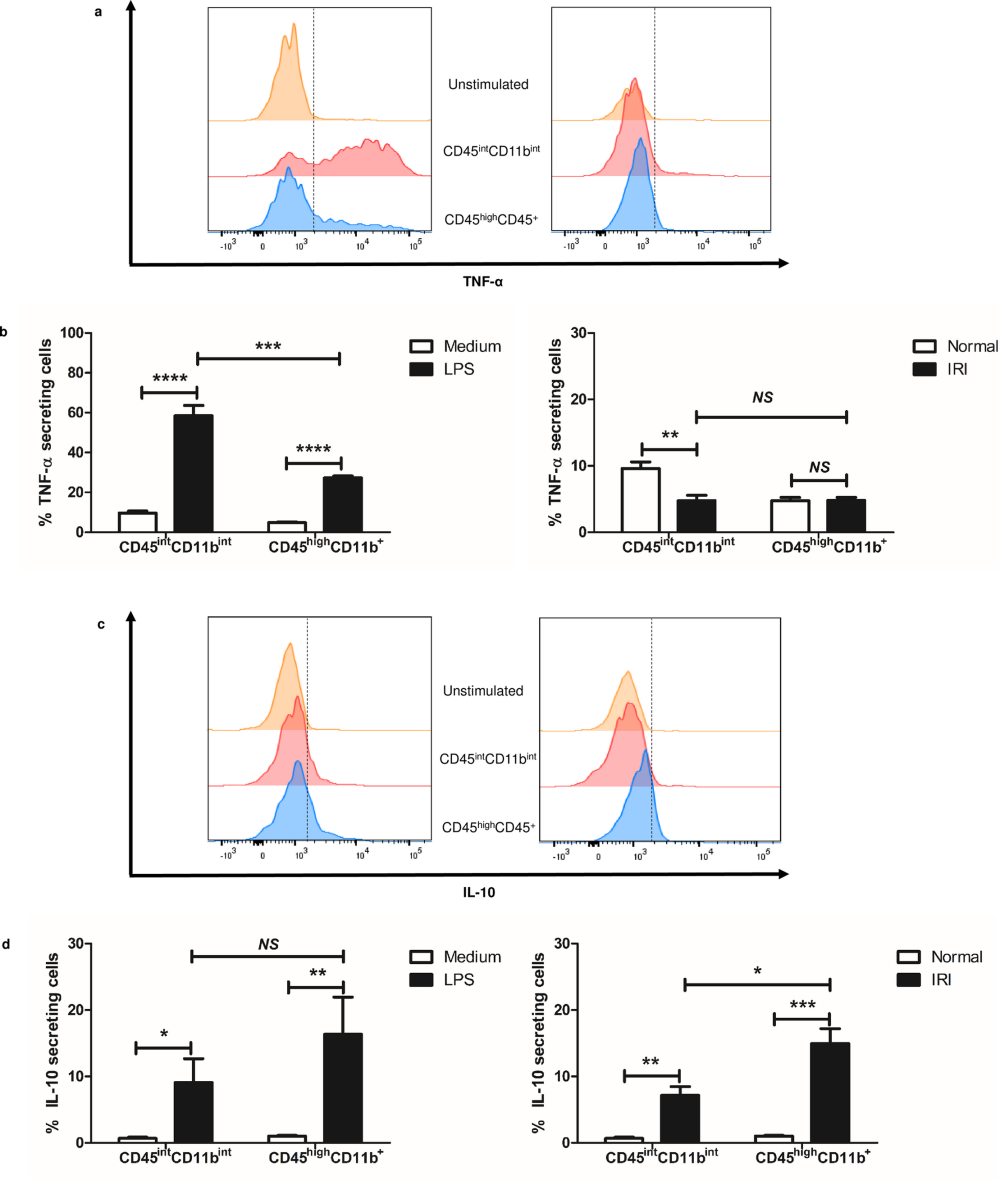
CD45^int^CD11b^int^ MPCs express distinct cytokines. Intracellular cytokine level in each MPC population was measured by flow cytometry. (a, c) Representative histograms show the expression level of TNF-α and IL-10 in each MPC population after LPS stimulation (left) or after ischemic injury (right). Yellow plot indicates the cytokine production of unstimulated MPCs from normal mice, being used as a control. Red plot and blue plot represent the cytokine expression by CD45^int^CD11b^int^ MPCs and CD45^high^CD11b^+^ MPCs, respectively. (b, d) Bar graph indicates the proportion of cells producing TNF-α and IL-10 within each MPC population after LPS stimulation (left) or after ischemic injury (right). Data are displayed as means ± SEM (n = 5/group). *P < 0.05; **P < 0.01; ***P < 0.001; ****P < 0.0001; *NS*, no statistically significant difference between groups.

To further delineate the functional differences between the CD45^int^CD11b^int^ and CD45^high^CD11b^+^ populations, we investigated their ability to produce signature pro- and anti-inflammatory cytokines using intracellular staining in response to LPS, a potent inducer of strong immune response, or after renal IRI (**Fig 7**). In the first set of experiments, we assessed their production of pro-inflammatory cytokines, TNF-α and IFN-γ. After 6 hours of LPS stimulation, a significantly higher percentage of CD45^int^CD11b^int^ MPCs expressed intracellular TNF-α compared to CD45^high^CD11b^+^ MPCs (58.4%±5.2% vs. 27.3%±0.9%, *P* < 0.001). Next, we analyzed their responses to IRI. Overall, IRI did not induce significant expression of TNF-α by either populations. Nonetheless, expression of TNF-α by CD45^int^CD11b^int^ MPCs in ischemic kidneys was lower than by control kidneys 24 hours after the surgery; however, the difference between the two was relatively small (9.0%±0.8% vs. 4.8%±0.8%, *P* < 0.01). Similarly, CD45^high^CD11b^+^ produced small amounts of TNF-α in response to IRI. The expression of IFN-γ was very low (less than 5.0%) and there was no significant difference between both MPC populations regardless of the LPS stimulation and ischemic injury (data not shown).

We next compared production of the anti-inflammatory cytokine, IL-10 and IL-4 by both populations in response to LPS and IRI. Our results show that CD45^int^CD11b^int^ MPCs produced less IL-10 than CD45^high^CD11b^+^ cells in response to LPS, but the difference was not statistically significant (7.3%±2.2% vs. 14.2%±3.8%, *P* = 0.16). A similar trend of lower IL-10 expression by CD45^int^CD11b^int^ MPCs was observed after IRI compared to the CD45^high^CD11b^+^ MPCs in post-ischemic mice (7.2%±1.3% vs. 14.9%±2.2%, *P* = 0.02). The expression of IL-4 was very low (less than 5.0%) and there was no significant difference between both MPC populations regardless of the LPS stimulation and ischemic injury (data not shown). Together, these data show that CD45^int^CD11b^int^ are potent producers of TNF-α in response to LPS stimulation, but less significant producers of IL-10 in response to IRI as compared to CD45^high^CD11b^+^.

### CD45^int^CD11b^int^ MPCs show different temporal frequency in comparison to CD45^high^CD11b^+^ population in response to kidney IRI

**Fig 8:**
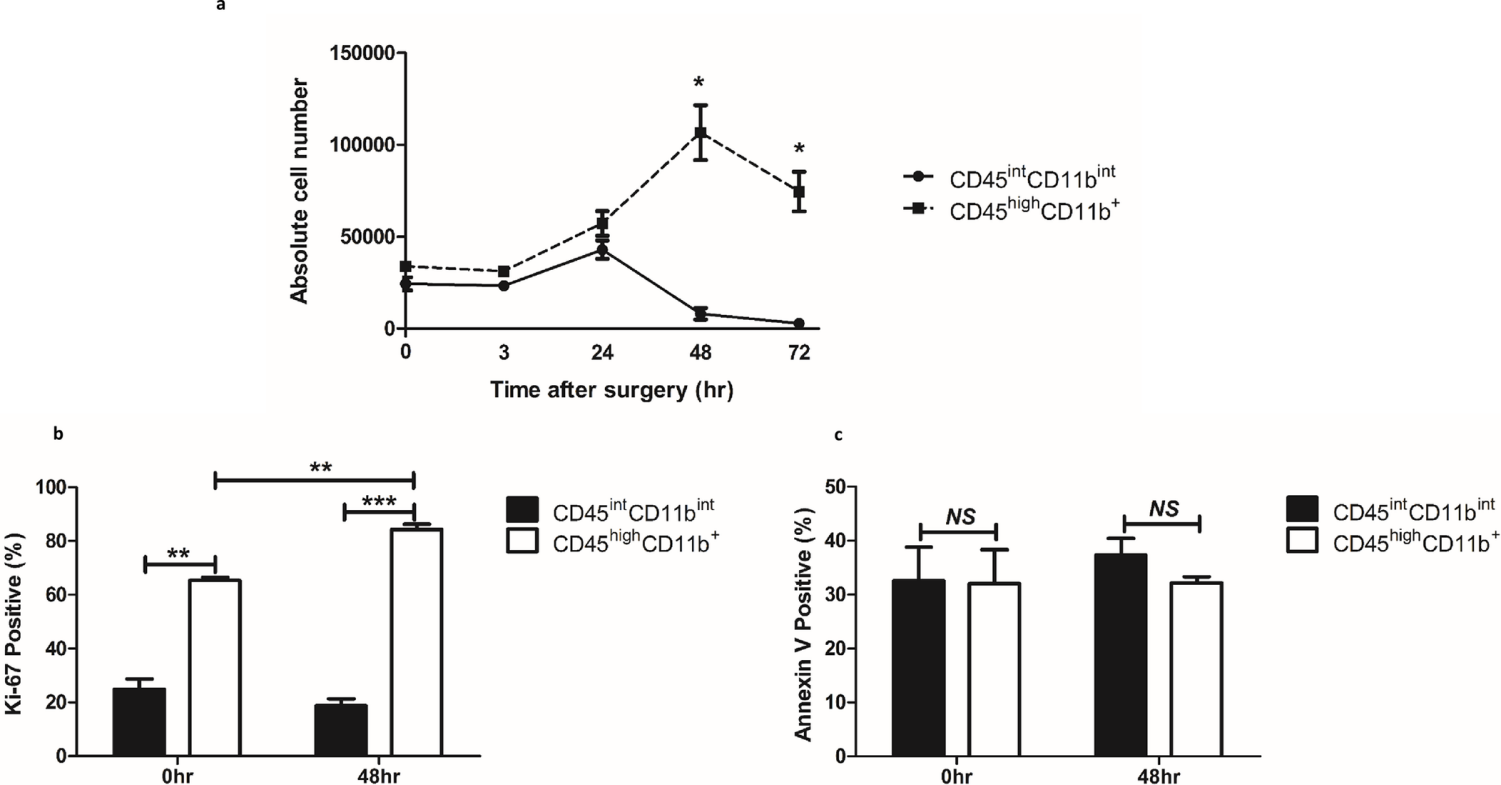
CD45^int^CD11b^int^ MPCs show distinct response to kidney ischemic reperfusion injury (IRI) compared to CD45^high^CD11b^+^ MPCs. (a) Serum creatinine levels were determined at baseline and 3, 24, 48, and 72 hours after kidney ischemia. Both populations show similar pattern until 24 hours after IRI. In contrast to the sustained increase of CD45^high^CD11b^+^ population at 48 hours, CD45^int^CD11b^int^ cells decrease significantly at 48 hours after IRI. The number of both population decrease at 72 hours after IRI. (b, c) Bar graph indicates percentage of cells expressing Ki-67 (b) or binding Annexin V (c) within each MPC population at baseline (left) or 48 hours after ischemic injury (right). Data are displayed as means ± SEM (n = 3/group). *P < 0.05; **P < 0.01; ***P < 0.001; *NS*, no statistically significant difference between groups.

Given the differences between the CD45^int^CD11b^int^ and the CD45^high^CD11b^+^ population in the normal mouse kidney, we used kinetics to evaluate responses of these two populations to IRI (Fig 8A). We performed bilateral kidney IRI using an established method [24, 25] and counted the absolute numbers of CD45^int^CD11b^int^ MPCs versus that of CD45^high^CD11b^+^ population at four time points (3, 24, 48 and 72 hours). CD45^int^CD11b^int^ and CD45^high^CD11b^+^ populations simultaneously started to increase 3 hours after IRI and the patterns remained similar 24 hours later. After that, the CD45^high^CD11b^+^ population continued to increase when measured at 48 hours and then declined slightly at 72 hours. On the other hand, CD45^int^CD11b^int^ MPCs abruptly declined 48 hours after IRI and remained significantly less than CD45^high^CD11b^+^ population at 72 hours (*P* = 0.03). Taken together, CD45^int^CD11b^int^ and CD45^high^CD11b^+^ have different kinetics in response to IRI.

To characterize mechanisms underlying the temporal changes seen in these two MPC subsets, we investigated their proliferation and apoptosis using Ki-67 and Annexin V assay, respectively [28, 29]. A significantly lower percentage of CD45^int^CD11b^int^ population expressed Ki-67 compared to CD45^high^CD11b^+^ cells at baseline (25.0%±3.8% vs. 65.4%±1.2%, *P* < 0.01) as well as at 48 hours after IRI (18.7%±2.6% vs. 84.4%±1.9%, *P* < 0.001) (**Fig 8B**). However, there was no difference in percentage of Annexin V positive cells between these two populations (**Fig 8C**). The different proliferation patterns of the two population partly explains significant cell number difference between two populations at 48 hours after IRI. Also, their distinct proliferation pattern emphasize further that CD45^int^CD11b^int^ and CD45^high^CD11b^+^ populations are discrete.

### Kidney CD45^int^CD11b^int^ population in humans

**Fig 9:**
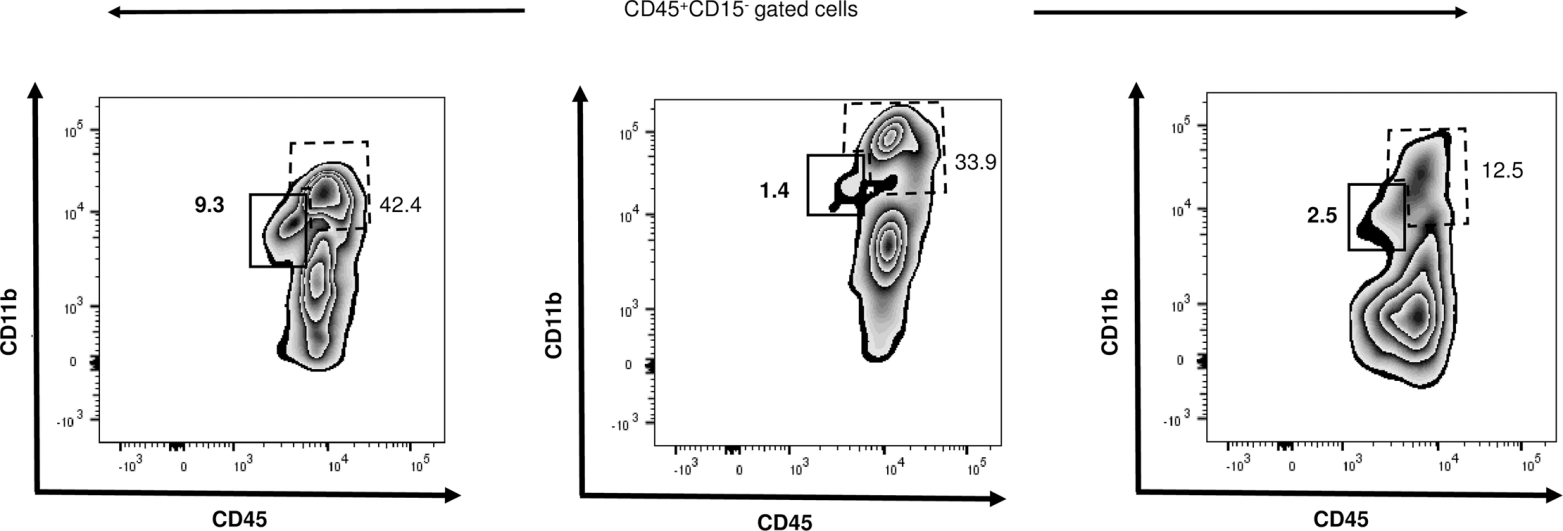
Detection of CD45^int^CD11b^int^ MPCs in human kidneys. Plots show CD45^+^CD15^-^ population in “normal” kidney tissue from three different de-identified individuals who underwent partial or total nephrectomies to treat renal cell carcinoma. CD45^int^CD11b^int^ MPCs are marked with solid line and CD45^high^CD11b^+^ population with dashed line. Numbers indicate the percentage of each population among CD45^+^ cells.

To assess whether CD45^int^CD11b^int^ MPCs are also present in human kidney, we isolated human KMNCs from carefully dissected “normal” tissue of three patients who underwent total or partial nephrectomy for RCC. Flow cytometric analysis of CD45^+^CD15^-^ population revealed that CD45^int^CD11b^int^ population were present in each of the specimens and their frequency varied from 1.4% to 9.3% of CD45^+^ cells (**Fig 9**).

## Discussion

In the current study, we uncovered the presence of a CD45^int^CD11b^int^ “intermediate MPCs” population in both murine and human kidneys. These cells are present in significantly high percentage in the kidney and to lesser extent in the heart but barely detectable in other lymphoid and non-lymphoid organs examined. The CD45^int^CD11b^int^ MPCs have a distinct surface phenotype, and their phagocytic function and cytokine production profiles differ from those of CD45^high^CD11b^+^ population. As compared to the CD45^high^CD11b^+^ population, these cells increase in mouse kidney immediately after ischemic AKI and then decrease 48 hours later, whereas the CD45^high^CD11b^+^ population continue to increase up to 48 hours after IRI before declining. Importantly, the CD45^int^CD11b^int^ population is found in human kidneys, showing their potential translational significance.

The high numbers of the kidney CD45^int^CD11b^int^ cells in the steady state as well as their distinct response pattern to IRI raise the possibility that the CD45^int^CD11b^int^ population serves different roles compared to CD45^high^CD11b^+^ MPCs. Based on our study and others, we can speculate on possible roles of this CD45^int^CD11b^int^ population. A study by Soos *et al*. showed that renal CX3CR1^+^ MPCs had significant co-expression of CD11c, F4/80, MHC II and FcR [30]. These markers were also expressed by the CD45^int^CD11b^int^ population, raising the possibility that CD45^int^CD11b^int^ and CX3CR1^+^ MPCs are related populations. CX3CR1^+^ MPCs have been described to constitute a contiguous network throughout the interstitial and mesangial spaces of the entire kidney, continually probing and sampling of the surrounding environment. The CX3CR1^+^ MPCs also showed an immature costimulatory function, but high competence in phagocytosis and immunoglobulin binding.

Cao *et al*. demonstrated that CD45^+^MHCII^+^F4/80^+^CD11c^+^ MPCs in kidney possessed higher phagocytic activity, but lower antigen-presenting capacity than other MPC populations, which implies that this F4/80^+^CD11c^+^ population has macrophage-like (rather than DC) functional characteristics in kidney [9]. When we applied the same gating strategy to our data, around 70–80% of F4/80^+^CD11c^+^ population were in CD45^int^CD11b^int^ gate while the remaining cells were in CD45^high^CD11b^+^ gate. In that study, the adoptive transfer of F4/80^+^CD11c^+^ population exacerbated renal injury in murine adriamycin nephrotoxicity (AN) model in spite of higher IL-10 expression in F4/80^+^CD11c^+^ cells. This discordant result suggests heterogeneity of F4/80^+^CD11c^+^ population and further sub-classification of F4/80^+^CD11c^+^ population based on their CD45 expression level may disclose the disparate functions of CD45^high^ and CD45^int^ populations among total F4/80^+^CD11c^+^ MPCs in murine AN.

Another study by Kawakami *et al*. showed that resident kidney MPCs can be classified into five discrete populations based on their CD11b and CD11c expression [12]. Among the five subpopulations in their study, CD11b^int^CD11c^int^ MPCs showed similar phenotypical characteristics to the CD45^int^CD11b^int^ population described in the present study, including higher expression of F4/80, MHCII and lower expression of Ly6C. The CD11b^int^CD11c^int^ subpopulation showed intermediate capacity of phagocytosis and T cell stimulation among other MPC subtypes. When naïve T cells were stimulated by the CD11b^int^CD11c^int^ population, they induced regulatory T cell differentiation, implying a possible role in immunological tolerance for this MPC subtype. However, when we applied the same gating strategy, it was difficult to identify five discrete MPC populations among CD45^**+**^Dump^**-**^ cells, making it hard to match the CD45^int^CD11b^int^ population directly to the CD11b^int^CD11c^int^ population of this study.

Li and colleagues have classified renal macrophages into two subsets, CD11b^low^F4/80^high^CX3CR1^high^ and CD11b^low^F4/80^high^CX3CR1^low^ cells [31]. The former population is considered a kidney resident MPCs and it shares similar phenotype with our CD45^int^CD11b^int^ population, as kidney resident MPCs. A more recent study, using parabiosis, also showed that F4/80^+^, CD11b^low^, MHCII^+^, CD11c^+^ macrophages have minimal exchange with the peripheral circulation, suggesting that this population is tissue-resident [32]. Interestingly, our CD45^int^CD11b^int^ population shares similar phenotype with our CD45^int^CD11b^int^ population, hence could also be tissue resident. Another study by Stamatiades *et al*.[33] showed that kidney tissue-resident macrophages, characterized as F4/80^high^, CX3CR1^high^, CD11^int^ population, serve immuno-surveillance role by taking up circulating immune complex (IC) via FcγRs. IC uptake by the tissue-resident macrophages triggers renal inflammation by increasing pro-inflammatory cytokine production and promoting subsequent neutrophil and monocyte infiltration. This study implies the role of kidney-resident macrophages in IC-mediated inflammatory disease, such as systemic lupus erythematosus. Based on the phenotypical overlap of macrophages between this study and our study, it is possible that the macrophage population described by this group is similar to what we describe, though functional response to IRI and LPS stimulation were not evaluated in the study [33]. Given the differences in phagocytic function we observed in CD45^int^CD11b^int^ MPCs compared to CD45^high^CD11b^**+**^ MPCs as well as differences in TNF-α production in LPS-stimulated kidney tissues and IL-10 production in post-ischemic kidney tissues of our study, it is possible that intermediate macrophages serve distinct roles from CD45^high^CD11b^**+**^ MPCs. More detailed functional studies are required to further understand the role of CD45^int^CD11b^int^ cells in different kidney diseases.

One of the most interesting findings of this present study is the high prevalence of the CD45^int^CD11b^int^ MPCs found in the kidney, but not in the other organs examined. There have been several studies about organ/tissue-specific cells including stem cells and immune cells [34, 35], which implied that certain cell types are composed of collection of phenotypically and functionally specialized sub-populations with different origins and tissue specificities. Future studies on the kidney CD45^int^CD11b^int^ population including their developmental origin as well as their interaction with the neighboring kidney structure will shed a light on their organ-specificity and their potential role in kidney diseases.

The impact of enzymes has been discussed for the proper single cell preparation of non-lymphoid organs. Despite risks of damage on membrane integrity and change in molecular expression [36, 37], many researchers continue using ED, because ED can bring effective disaggregation of their target tissues with improved yield. The recently published study by Williams *et al*. [38], showed that the effect of ED was greater for the isolation of F4/80 expressing cells, which seems in accord with our CD45^int^CD11b^int^ MPC population. Our finding on the effects of ED for the isolation of CD45^int^CD11b^int^ population implies that ED is recommended over MD for future studies on kidney MPC population.

Furthermore, our data show that FcR blocking can significantly reduce the chance of misrepresenting CD45^int^CD11b^int^ MPCs as T cells. These two populations have very similar light scatter signals (**S6 Fig**) and CD45^int^CD11b^int^ population demonstrated FcR-mediated false positivity with specific T cell antibodies (**S7 Fig**). We could not explain the reason why the CD45^int^CD11b^int^ population showed intense fluorescent signal from anti-TCRβ and CD8β mAbs but not from other T cell antibodies. A study by Iwasaki *et al*. implies possible mechanism of this phenomenon through trogocytosis, a recently recognized intercellular communication process among immune cells [39]. This study demonstrates CD8 expression on the surface of human monocytes, which results from CD8 translocation from T cells to conjugated monocytes mediated by CD8 mAb and FcR on monocytes, called FcR-mediated trogocytosis. This study suggests more caution on analyzing flow cytometry data to rule out any false positive results mediated by trogocytosis, which can be accomplished by proper usage of FcR blockers.

It is unclear whether the kidney CD45^int^CD11b^int^ population has been already studied by others due to multiple gating strategies employed by different kidney studies. However, sub-classification of kidney MPC population using both CD45 and CD11b expression level is a novel approach and this strategy will provide help on further classification of kidney MPC subpopulation based on their complexity and absence of surrogate markers in kidney MPC studies. It should also be noted that though we studied human kidney tissue that was grossly and histologically normal tissue from nephrectomy samples conducted to treat RCC, the tissue may not be totally normal since they were near the cancer. Nevertheless, these data still demonstrate the presence of CD45^int^CD11b^int^ in human kidney.

In summary, our data indicate the presence of a newly demonstrated subset of kidney MPCs in mice and humans which can be characterized as CD45^int^CD11b^int^F4/80^+^MHCII^+^CX3CR1^+^Ly6C^-^ “intermediate MPCs”. Future studies are required to understand functional attributes of this intermediate MPC population in mouse and human and its relationship to other MPC subsets during health and disease.

## Supporting information

S1 Fig Both enzymatic digestion (ED) and mechanical digestion (MD) show similar viability for isolation of kidney mononuclear cells. (a) Representative 7-AAD vs. SSC-A plots of kidney mononuclear cells show no difference of cell viability. Numbers on plots represent proportions of 7-AAD negative cells. (b) Graphs show the percentages of viable cells in each digestive methods (ED, 95.2%±0.8% vs. MD, 93.6%±1.4%). Data are displayed as means ± SEM. (n = 3/group).
(PDF)

S2 Fig Gating strategy for assessing CD45^int^CD11b^int^ and CD45^high^CD11b^+^ population in the mouse kidney. Representative plots show the gating hierarchy to study CD45^int^CD11b^int^ and CD45^high^CD11b^+^ population in mouse kidney with enzymatic digestion (ED) (a) and mechanical digestion (MD) alone (b). The isolation of CD45^int^CD11b^int^ population is more effective with ED compared to MD alone.
(PDF)

S3 Fig Gating strategy for assessing TCRβ^+^CD4^-^CD8α^-^β^+^ cells in the mouse kidney. Representative plots show the gating hierarchy for TCRβ^+^CD4^-^CD8α^-^β^+^ population in mouse kidney with enzymatic digestion (ED) (a) and mechanical digestion alone (MD) (b). The pattern of surface marker expression is consistent in both ED and MD method.
(PDF)

S4 Fig CD45^int^ population does not express lineage markers of lymphocytes, neutrophils or NK/NKT cells. Representative plots show the expression of lineage markers in CD45^int^ (Filled) and CD45^high^ (Dashed) population.
(PDF)

S5 Fig Collagenase exposure does not decrease the surface expression of CD45 and CD11b. (a) Representative CD45 vs. CD11b plots of bone marrow macrophages after incubation with collagenase (left) or 5% RPMI media (right). Numbers on plots represent the percentage of CD45^+^CD11b^+^ population among the singlets. (b) Graphs show the mean fluorescence intensity of each marker in bone marrow macrophages. Data are displayed as means ± SEM (n = 3/group). ***P < 0.001; *NS*, no statistically significant difference between groups; MFI, mean fluorescence intensity.
(PDF)

S6 Fig CD45^int^CD11b^int^ MPCs have lower forward- and side-scatter light signals compared to CD45^high^CD11b^+^ population sharing similar location with lymphocytes in light scatter gate. Backgating analysis of each immune cell population was used to compare the relative location of CD45^int^CD11b^int^ MPCs (green), CD45^high^CD11b^+^ MPCs (red), Ly6G^+^ granulocytes (blue) and lymphocytes (black), which shows that CD45^int^CD11b^int^ MPCs have lymphocyte-like light scatter signals.
(PDF)

S7 Fig Fc receptor blockade prevents the binding of TCRβ and CD8β antibodies on the CD45^int^ population. (a) Histogram shows that CD45^int^ cells bind TCRβ and CD8β specific antibodies, but not CD8α or CD4 without Fc receptor blocking. (b) The positive signals from TCRβ and CD8β disappear after incubation with Fc receptor blockers. Graphs are from one of three experiments with similar results. Blue-dashed plot represents isotype control and red-filled plot indicates signals from CD45^int^ population. FcR, Fc receptor.
(PDF)
