# Correction: Characterization of kidney CD45^int^CD11b^int^F4/80^+^MHCII^+^CX3CR1^+^Ly6C^-^ "intermediate mononuclear phagocytic cells"

**DOI:** 10.1371/journal.pone.0200056

**Published:** 2018-06-27

**Authors:** The PLOS ONE Staff

The first author's name should be written as Sul A Lee. The publisher apologizes for the error.
