# Supplementary figures and images for: Characterization of kidney CD45intCD11bintF4/80+MHCII+CX3CR1+Ly6C- “intermediate mononuclear phagocytic cells”
Source: PLoS One. 2018 Jun 1;13(6):e0198608. doi: 10.1371/journal.pone.0198608 (PMC5983557; doi:10.1371/journal.pone.0198608)

Supplementary figure 1

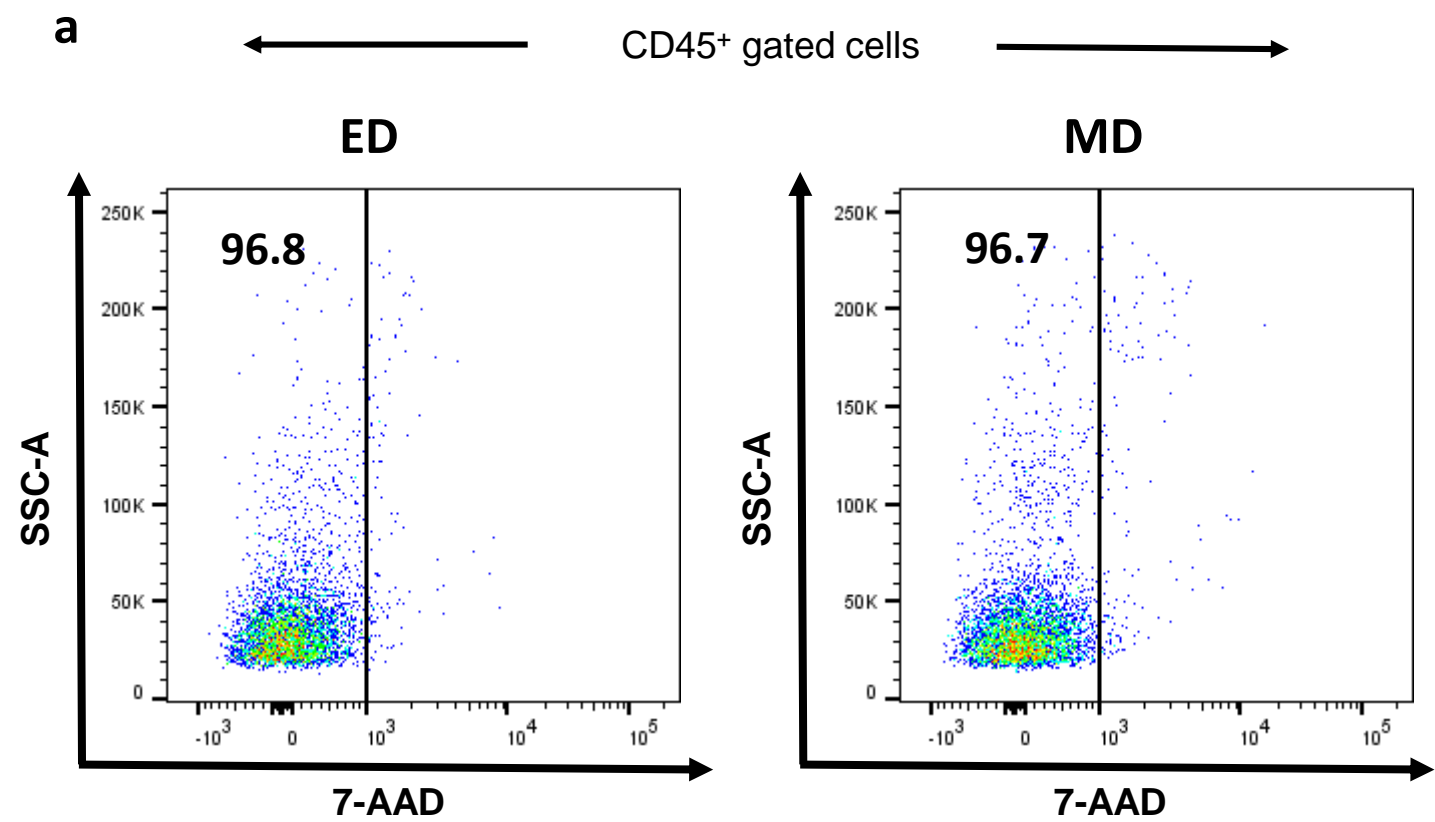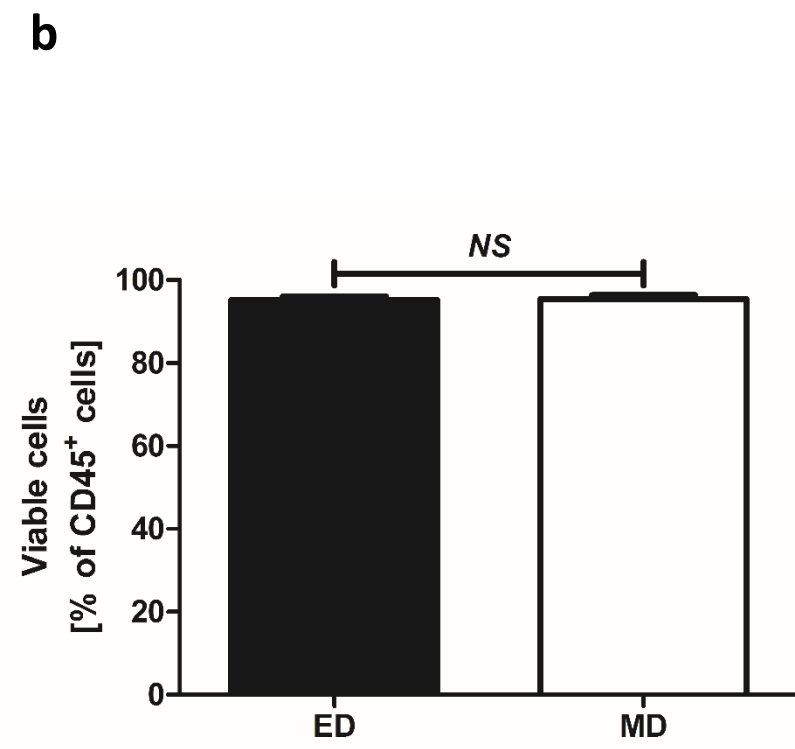

Supplement: S1 Fig — (a) Representative 7-AAD vs. SSC-A plots of kidney mononuclear cells show no difference of cell viability. Numbers on plots represent proportions of 7-AAD negative cells. (b) Graphs show the percentages of viable cells in each digestive methods (ED, 95.2%±0.8% vs. MD, 93.6%±1.4%). Data are displayed as means ± SEM. (n = 3/group). (PDF) [file pone.0198608.s001.pdf]

Supplementary figure 2

**a**

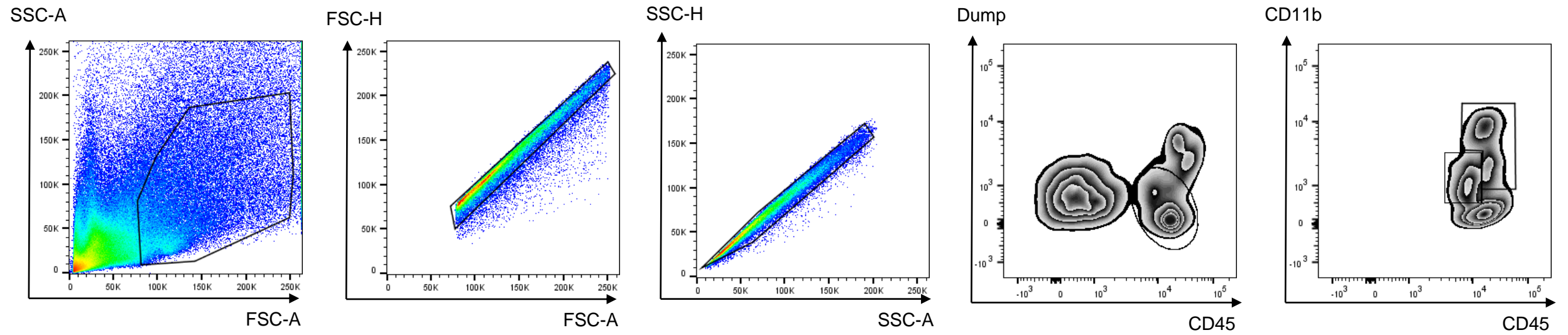

**b**

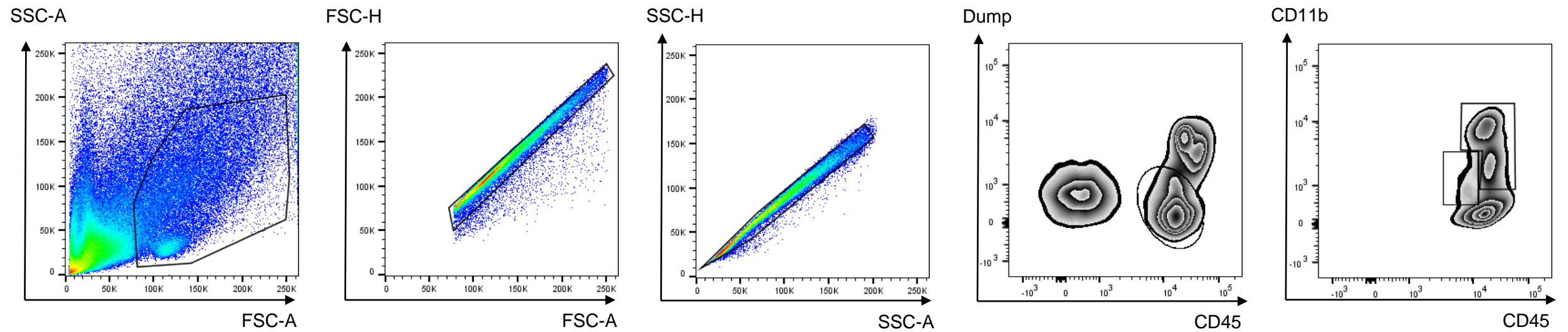

Supplement: S2 Fig — Representative plots show the gating hierarchy to study CD45intCD11bint and CD45highCD11b+ population in mouse kidney with enzymatic digestion (ED) (a) and mechanical digestion (MD) alone (b). The isolation of CD45intCD11bint population is more effective with ED compared to MD alone. (PDF) [file pone.0198608.s002.pdf]

Supplementary figure 3

**a**

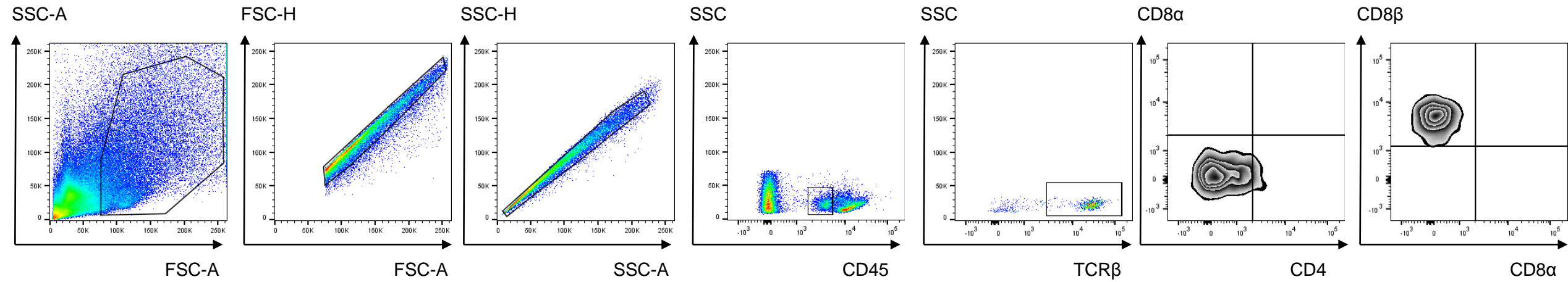

**b**

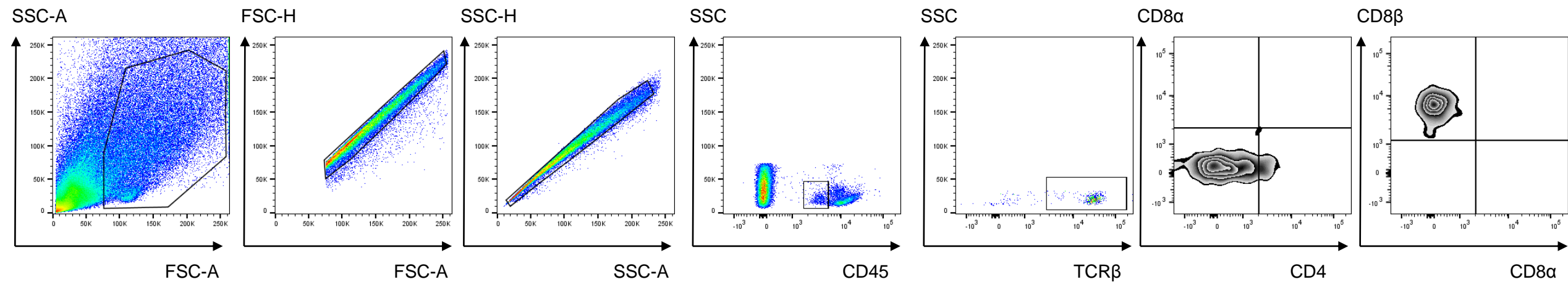

Supplement: S3 Fig — Representative plots show the gating hierarchy for TCRβ+CD4-CD8α-β+ population in mouse kidney with enzymatic digestion (ED) (a) and mechanical digestion alone (MD) (b). The pattern of surface marker expression is consistent in both ED and MD method. (PDF) [file pone.0198608.s003.pdf]

Supplementary figure 4

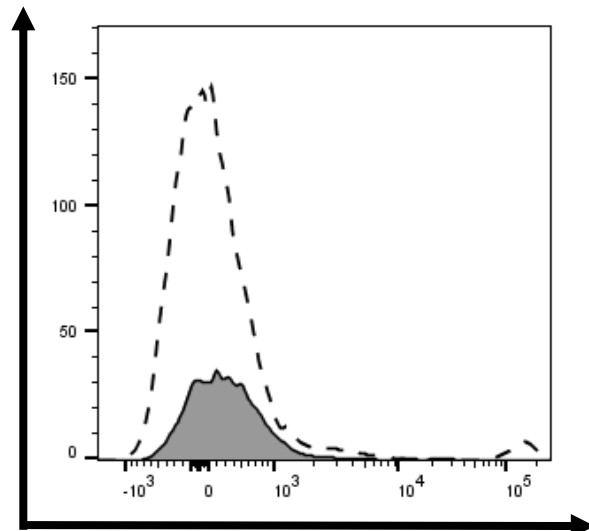

Ly6G

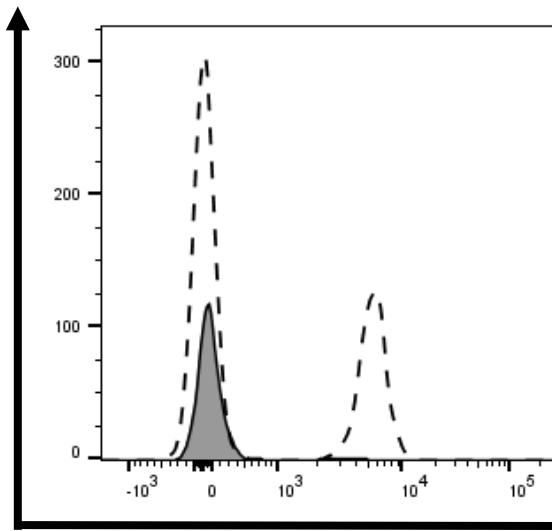

CD19

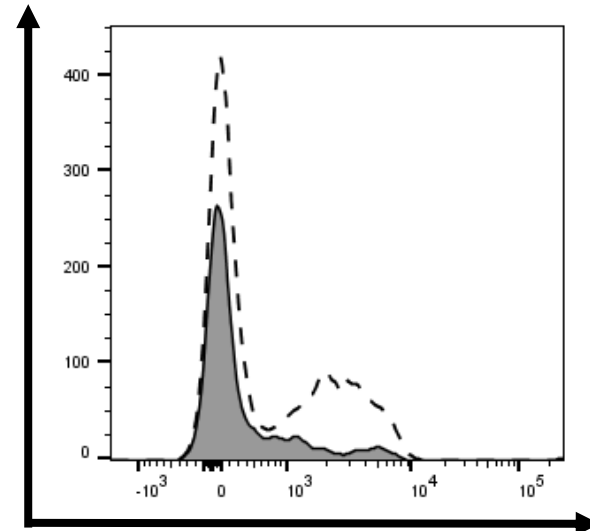

TCR $\beta$

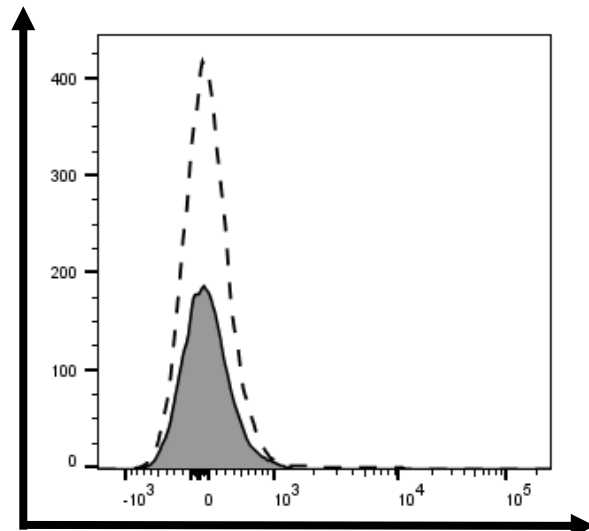

TCR $\gamma\delta$

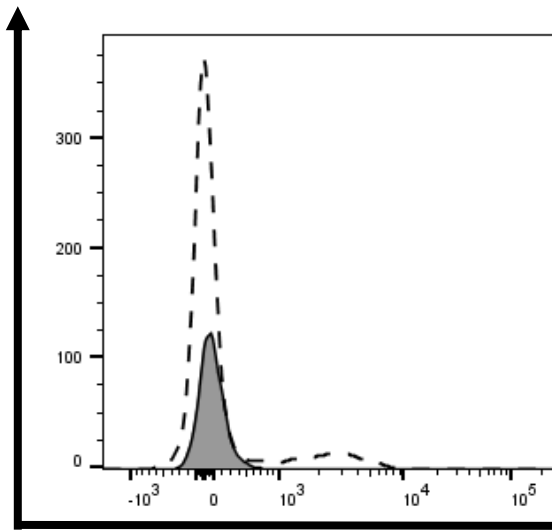

NK1.1

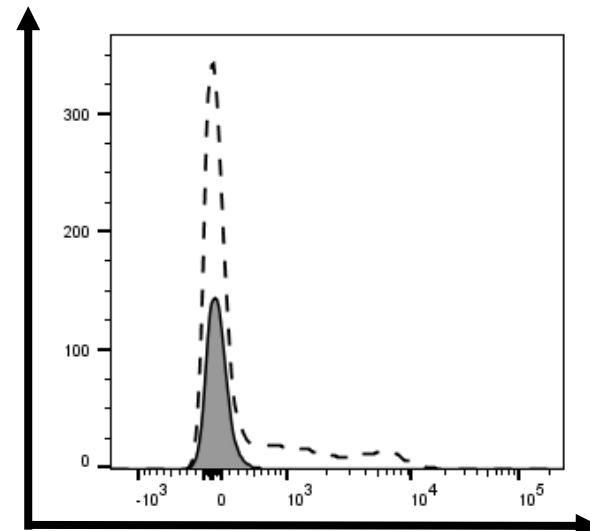

CD49b

Supplement: S4 Fig — Representative plots show the expression of lineage markers in CD45int (Filled) and CD45high (Dashed) population. (PDF) [file pone.0198608.s004.pdf]

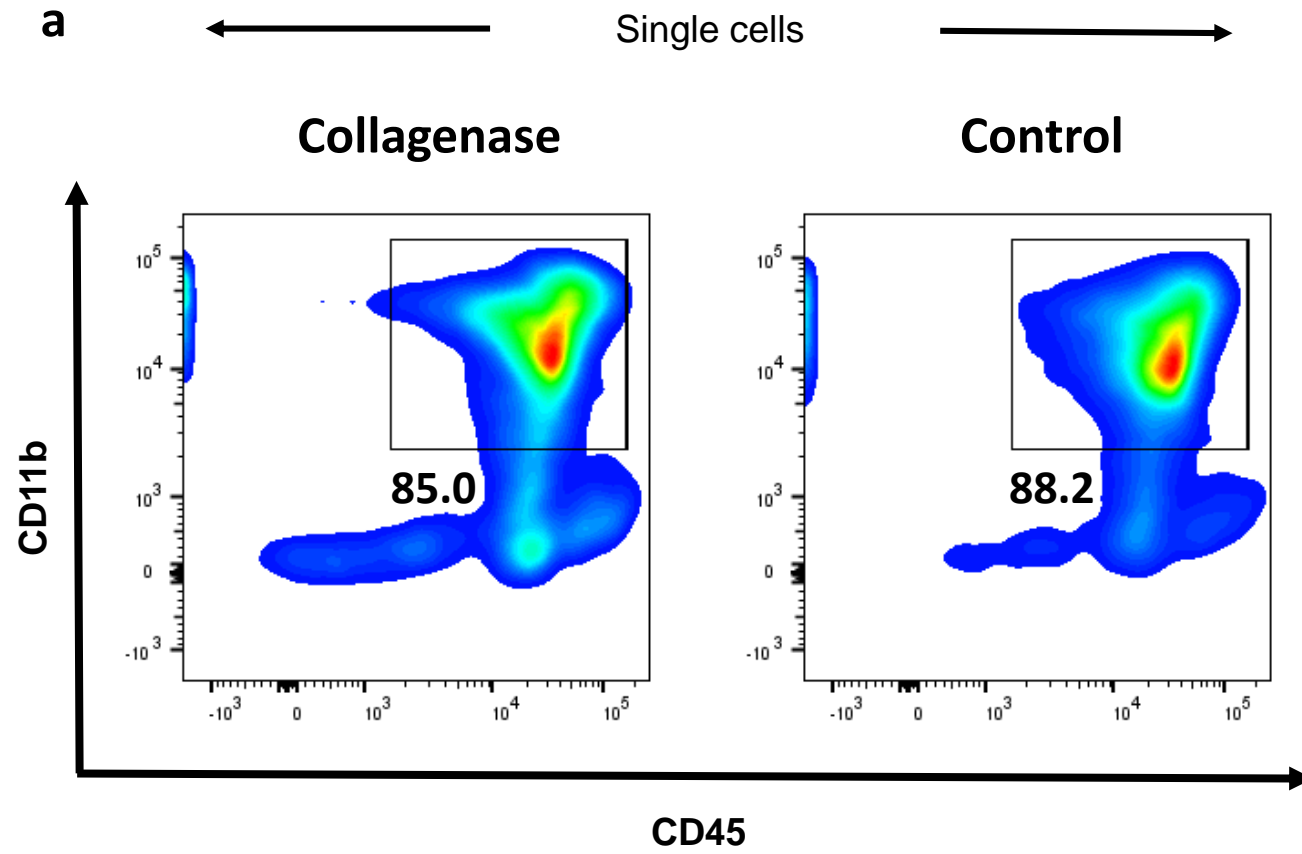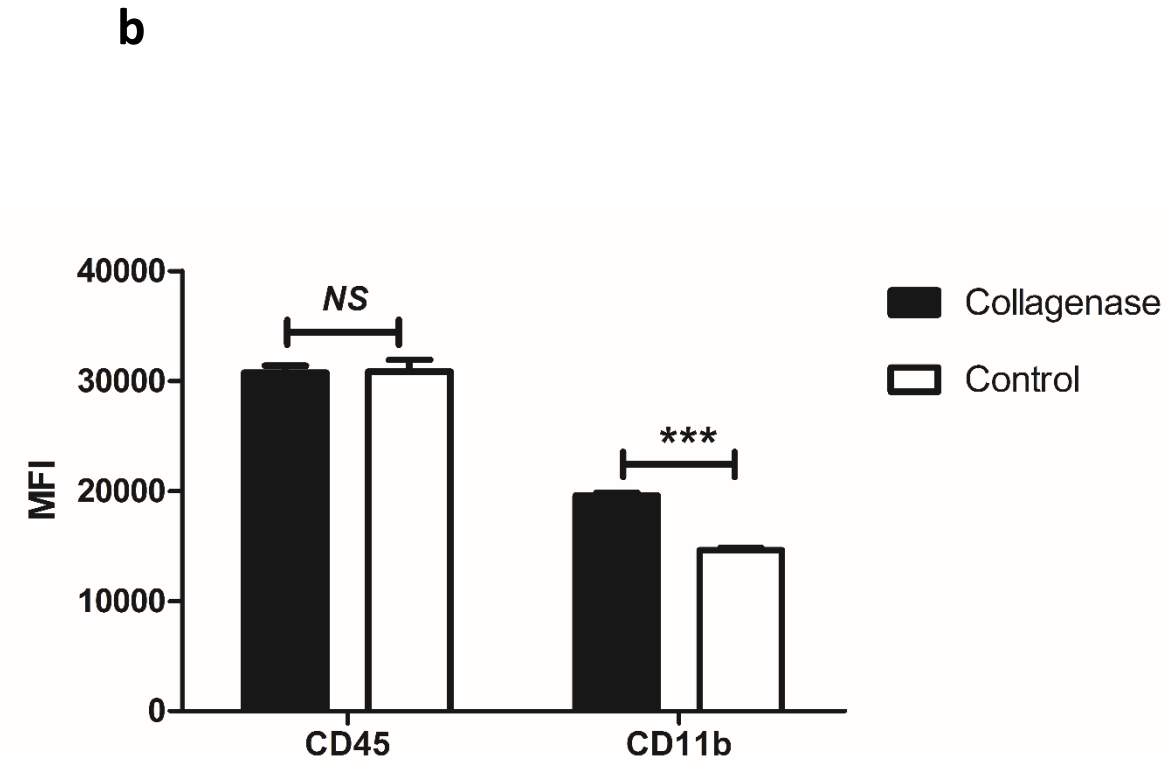

Supplement: S5 Fig — (a) Representative CD45 vs. CD11b plots of bone marrow macrophages after incubation with collagenase (left) or 5% RPMI media (right). Numbers on plots represent the percentage of CD45+CD11b+ population among the singlets. (b) Graphs show the mean fluorescence intensity of each marker in bone marrow macrophages. Data are displayed as means ± SEM (n = 3/group). ***P < 0.001; NS, no statistically significant difference between groups; MFI, mean fluorescence intensity. (PDF) [file pone.0198608.s005.pdf]

Supplementary figure 6

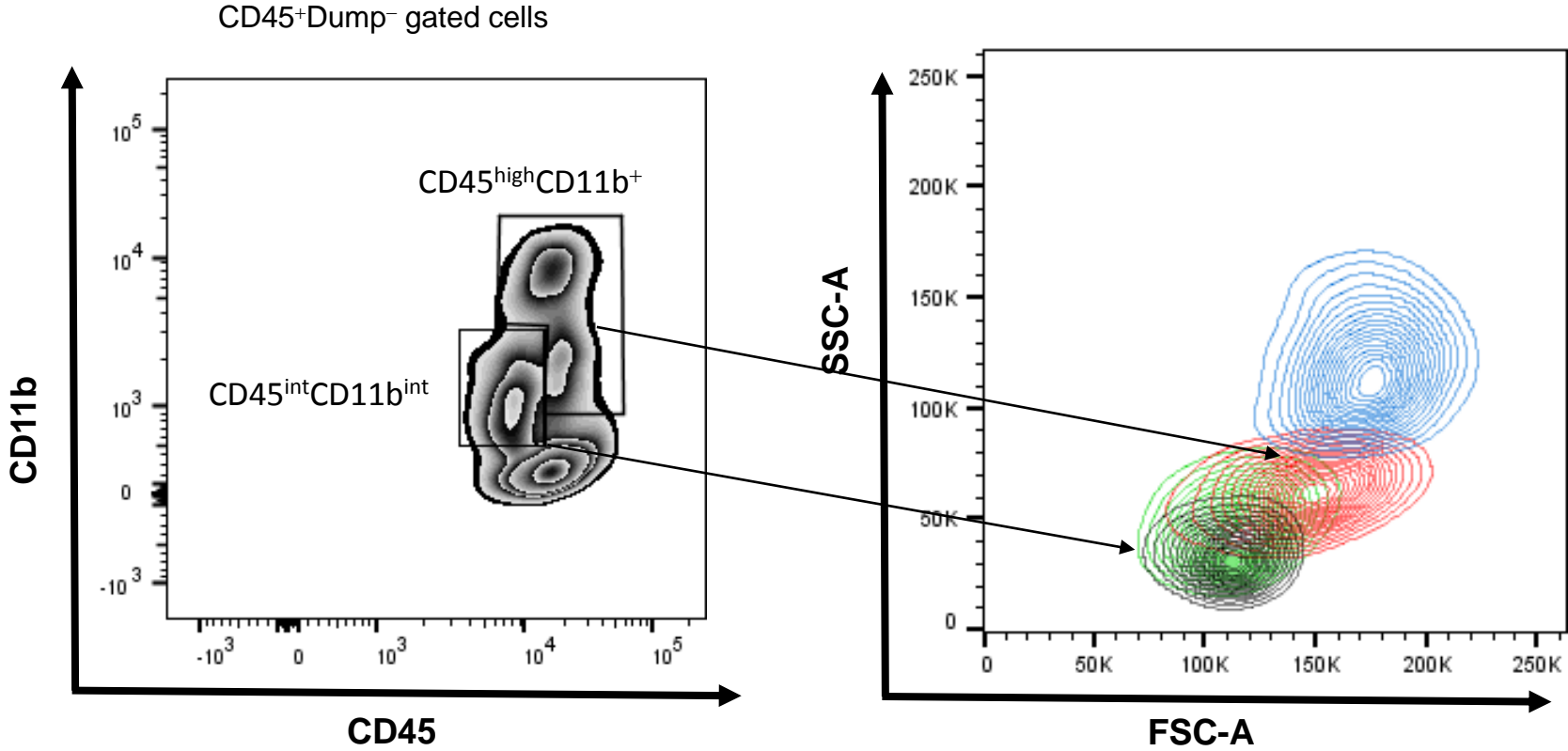

Supplement: S6 Fig — Backgating analysis of each immune cell population was used to compare the relative location of CD45intCD11bint MPCs (green), CD45highCD11b+ MPCs (red), Ly6G+ granulocytes (blue) and lymphocytes (black), which shows that CD45intCD11bint MPCs have lymphocyte-like light scatter signals. (PDF) [file pone.0198608.s006.pdf]

**a**

Without FcR blocker

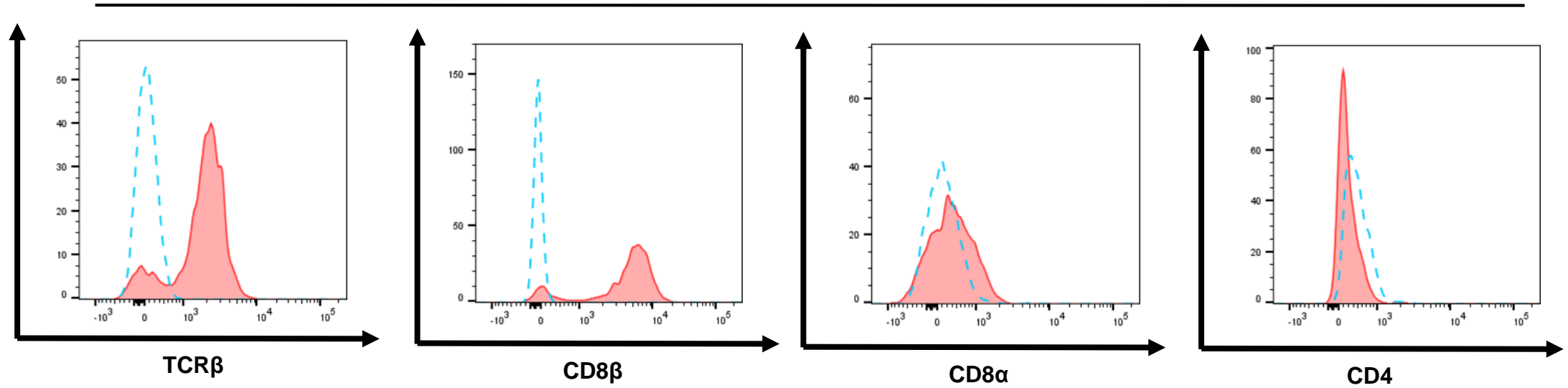

**b**

With FcR blocker

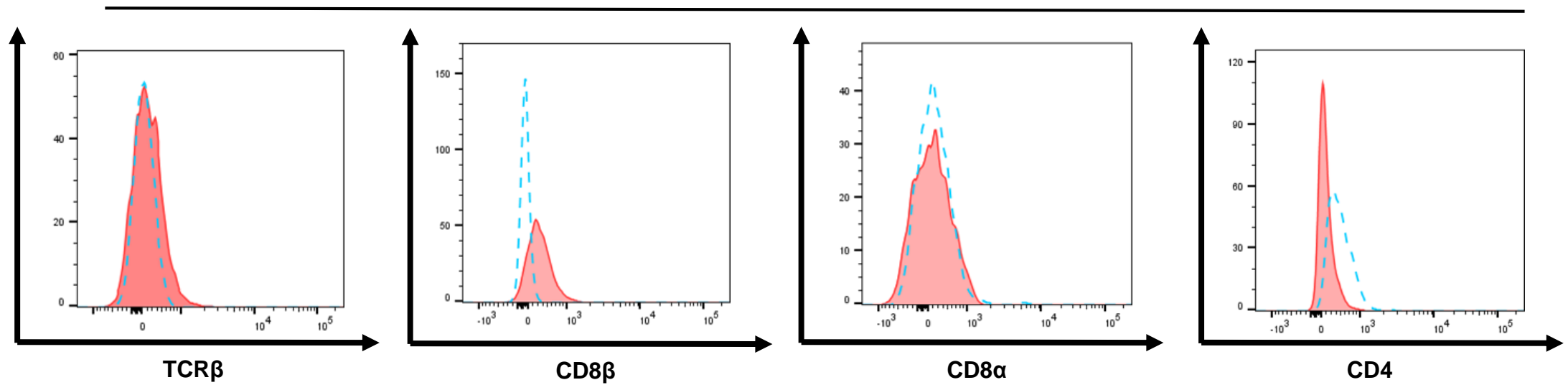

Supplement: S7 Fig — (a) Histogram shows that CD45int cells bind TCRβ and CD8β specific antibodies, but not CD8α or CD4 without Fc receptor blocking. (b) The positive signals from TCRβ and CD8β disappear after incubation with Fc receptor blockers. Graphs are from one of three experiments with similar results. Blue-dashed plot represents isotype control and red-filled plot indicates signals from CD45int population. FcR, Fc receptor. (PDF) [file pone.0198608.s007.pdf]
